# Quantitation of Key Antioxidants and Their Contribution to the Oxidative Stability of Beer

**DOI:** 10.1021/acs.jafc.4c01000

**Published:** 2024-07-16

**Authors:** Stefan Spreng, Corinna Dawid, Andreas Dunkel, Thomas Hofmann

**Affiliations:** † Chair of Food Chemistry and Molecular and Sensory Science, Technical University of Munich, Lise-Meitner-Str. 34, D-85354 Freising, Germany; ‡ Bavarian Center for Biomolecular Mass Spectrometry, Gregor-Mendel-Straße 4, D-85354 Freising, Germany; § Leibniz-Institute for Food Systems Biology at the Technical University of Munich, Lise-Meitner Str. 34, D-85354 Freising, Germany

**Keywords:** antioxidants, beer, flavor stability, hordatines, LC-MS/MS, phenols, tachioside

## Abstract

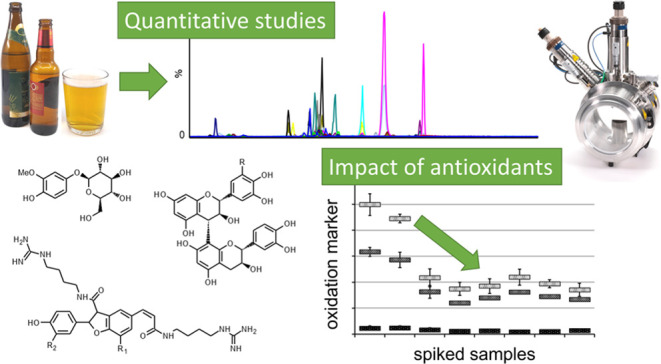

A sensitive high-performance liquid chromatography–triple quadrupole mass spectrometry (HPLC-MS/MS_MRM_) method, leveraging both technique and internal calibration, was developed for the simultaneous and comprehensive quantitative analysis of 46 antioxidants and antioxidant precursors in different beer types without any cleanup procedure. Combined with their *in vitro* antioxidant activity, a dose-activity estimation exposed a group of 10 key antioxidants, namely, tryptophan, tyrosine, hordatine A, hordatine B, procyanidin B_3_, prodelphinidin B_3_, tachioside (3-methoxy-4-hydroxyphenyl-β-d-glucopyranoside), (+)-catechin, tyrosol, and ferulic acid. To study the effect of antioxidants in spiking and aging studies, another liquid chromatography-MS (LC-MS)-based method was developed, monitoring markers for oxidation in beer. A positive effect of the antioxidants on the flavor stability at naturally relevant concentrations was shown by a slowing of oxygen-dependent aging reactions highlighted in beer storage trials under oxygen atmosphere. Thereby, a doubling of the natural concentration of all investigated antioxidants in beer revealed a limit inhibition of 67% on the degradation of *cis-*isocohumulone to hydroxy-*cis-*alloisocohumulone.

## Introduction

**Figure 1:**
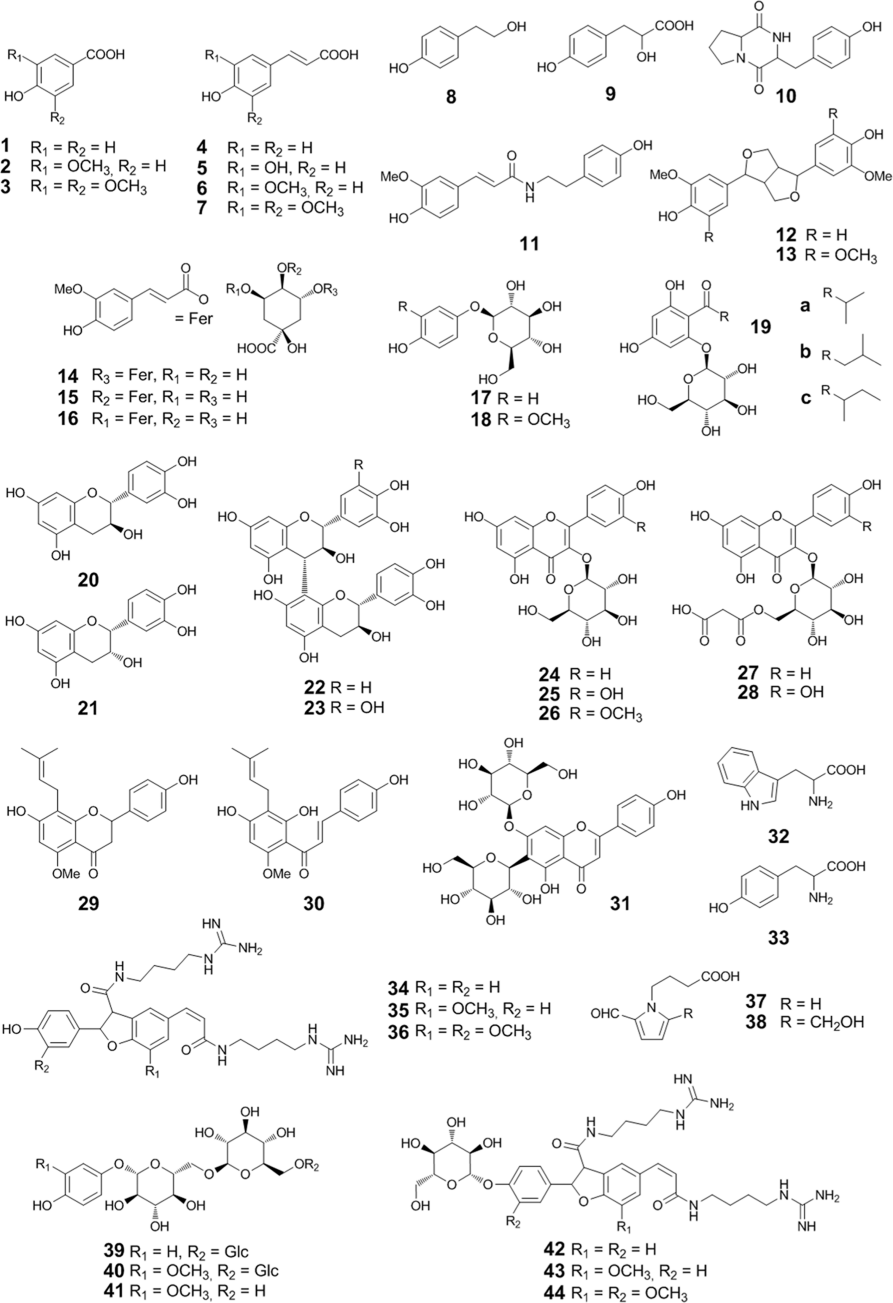
Chemical structures of antioxidants and antioxidant precursors investigated in beer: *p*-hydroxybenzoic acid (**1**), vanillic acid (**2**), syringic acid (**3**), *p*-coumaric acid (**4**), caffeic acid (**5**), ferulic acid (**6**), sinapic acid (**7**), tyrosol (**8**), *p*-hydroxyphenyllactic acid (**9**), *cyclo* (Pro-Tyr) (**10**), *N-*feruloyltyramine (**11**), pinoresinol (**12**), syringaresinol (**13**), 5-feruloylquinic acid (**14**), 4-feruloylquinic acid (**15**), 3-feruloylquinic acid (**16**), arbutin (**17**), tachioside (**18**), *co*-multifidol glucoside (**19a**), *n*-multifidol glucoside (**19b**), *ad*-multifidol glucoside (**19c**), (+)-catechin (**20**), (−)-epicatechin (**21**), procyanidin B_3_ (**22**), prodelphinidin B_3_ (**23**), kaempferol glucoside (**24**), quercetin glucoside (**25**), isorhamnetin glucoside (**26**), kaempferol malonylglucoside (**27**), quercetin malonylglucoside (**28**), isoxanthohumol (**29**), xanthohumol (**30**), saponarin (**31**), tryptophan (**32**), tyrosine (**33**), hordatine A (**34**), hordatine B (**35**), hordatine C (**36**), 4-(2-formylpyrrol-1-yl)butyric acid (**37**), 4-[2-formyl-5-(hydroxymethyl)pyrrol-1-yl]butyric acid (**38**), 4-hydroxyphenyl-β-d-glucopyranosyl-(1 → 6)-β-d-glucopyranosyl-(1 → 6)-β-d-glucopyranoside (arbutintrioside, **39**), 4-hydroxy-3-methoxyphenyl-β-d-glucopyranosyl-(1 → 6)-β-d-glucopyranosyl-(1 → 6)-β-d-glucopyranoside (tachiotrioside, **40**), 4-hydroxy-3-methoxyphenyl-β-d-glucopyranosyl-(1 → 6)-β-d-glucopyranoside (tachiodioside, **41**), hordatine A glucoside (**42**), hordatine B glucoside (**43**), and hordatine C glucoside (**44**).

Due to its well-balanced aroma and taste as well as its refreshing character, beer is one of the most consumed alcoholic beverages throughout the world. Since the sensory profile of beer undergoes unwanted changes during storage, flavor stability is the major challenge to increase the shelf life of beer, besides the haze stability.^1,2^ Among a variety of compounds being linked to the flavor quality of beer,^1^ Strecker aldehydes have the highest impact on the aroma alteration of lager-type beer.^3−6^ The unstable iso-α-acids, principal bitter constituents in fresh beer, are, however, responsible for the most important changes in taste.^7,8^ The main degradation pathways were found to be cyclization reactions as well as the formation of hydroperoxides and hydroxides,^9,10^ mainly evoked by oxygen-mediated mechanisms. Thus, antioxidants seem to be able to increase the shelf life of beer.^1^ Due to national restrictions and a trend toward untreated products, the application of food additives is limited. As a consequence, studies have focused on naturally occurring antioxidants such as phenolics.^11−13^ Recent application of an activity-guided fractionation approach succeeded in mapping and identifying molecular determinants of the antioxidant activity of beer.^14^ Among these molecules, a series of phenolic compounds known from beer as well as previously unknown constituents like phenolglucoside **18** or hordatines (**34**–**36**) were discovered (Figure 1), which have been found to originate from the brewing malt, being partially released during fermentation from their precursors.^15^

A series of attempts have been made in recent years to quantitatively determine the antioxidant content in different beer types, especially hydroxybenzoic acid (**1**–**3**) and hydroxycinnamic acid (**4**–**7**) as well as flavan-3-ol derivatives (**20**–**23**).^12^ The majority of quantitative analysis was based on the use of high-performance liquid chromatography using electrochemical detection or ultraviolet (UV) detection,^16−21^ but recently also tandem mass spectrometry (MS/MS) detection,^22^ predominantly after extraction with ethyl acetate.^16,18−21^ However, the vast number of analytes requires a precise method with a high selectivity to avoid misidentification but also with a high throughput and easy sample preparation to be adequate for comprehensive studies. Moreover, no study showed convincingly an effect of single antioxidants on oxidative aging reactions in relevant concentrations. Only a decelerating effect of **6** and **20** on the degradation of the iso-α-acids was reported; however, the antioxidants were added in amounts exceeding the natural concentration by far.^23^ Therefore, the objectives of the present study were the following: (i) Development of a suitable high-performance liquid chromatography-MS/MS_MRM_ (HPLC-MS/MS_MRM_) method to quantitate the antioxidants in different beer types and to reveal the most important antioxidants through dose-activity estimations by calculating activity values. (ii) The verification of the antioxidants’ impact on the flavor stability of beer in natural amounts. This should be achieved by (ii,a) revealing appropriate analytical oxidation markers from untargeted analysis of beer storage trials forced by an oxygen atmosphere, as well as by (ii,b) spiking and storage trials, to analyze the effect on relevant aging reactions in beer.

## Materials and Methods

### Chemicals

The following compounds were obtained commercially: 2,2′-azo-bis(2-methylpropionamidine) (AAPH), fluorescein sodium salt, (±)-6-hydroxy-2,5,7,8-tetramethylchromane-2-carboxylic acid (Trolox), 2,2′-azinobis(3-ethylbenzothiazoline-6-sulfonic acid) diammonium salt (ABTS), acetic acid, (+)-catechin hydrate, disodium hydrogen phosphate, (−)-epicatechin, ethylenediaminetetraacetic acid hemoglobine, hydrogen peroxide, iron(II) sulfate heptahydrate, peroxidase from horseradish, linoleic acid, d(+)-maltose monohydrate, 4-methoxyhydrochinone, *p*-hydroxyphenyllactic acid, pinoresinol, d(−)-ribose, silver(I) carbonate, sodium tetraborate, syringic acid, tetramethylammonium hydroxide pentahydrate, 3,4,5-trimethoxybenzoic acid, triton X-100, l-tryptophan, Tween 20, l-tyrosine, tyrosol (Sigma-Aldrich, Steinheim, Germany); caffeic acid, formic acid (98–100%), hydrochloric acid (32%), *p*-hydroxybenzoic acid, potassium dihydrogen phosphate, potassium hydroxide, 2-propanol, sodium hydroxide (Merck, Darmstadt, Germany); ammonium acetate (5 M in water), ferulic acid, fluorescein, *p*-coumaric acid, sinapic acid, vanillic acid (Fluka, Neu-Ulm, Germany); *o*-coumaric acid (Roth, Karlsruhe, Germany); D_2_O, methanol-*d*_4_ (Euriso-Top, Saarbrücken, Germany); sodium hydroxide (Riedel-de-Haen, Seelze, Germany); *cyclo*(l-Pro-l-Tyr) (Bachem, Weil am Rhein, Germany); benzoylleucomethylene blue (TCI Europe, Zwijndrecht, Belgium); apigenin, dihydrorobinetin, isorhamntein-3-*O*-glucoside (Extrasynthèse, Genay, France); l-tryptophan (indol-*d*_5_), l-tyrosine (cycle-*d*_4_) (Cambridge Isotope Laboratories, Andover). Feruloylquinic acid (isolated from green coffee) and xanthohumol and isoxanthohumol (isolated from hops and hop products) were obtained from the Chair of Food Chemistry and Molecular Sensory Science (Freising, Germany). Water for high-performance liquid chromatography (HPLC) separation was purified by means of a Milli-Q water advantage A 10 water system (Millipore, Molsheim, France). Solvents were of HPLC grade (J.T. Baker, Deventer, the Netherlands) and ethyl acetate was purified by distillation in vacuum at 40 °C. Beer samples, listed in Table S1 in the Supporting Information, were purchased from the German retail market.

### Preparation of Reference Compounds

Following the protocols reported recently,^14^ compounds **13**, **18**, **19a**–**c**, **24**–**28**, **31**, and **34**–**38** were isolated from beer and a hop polyphenol extract. Compounds **39**–**44** were isolated from barley as very recently described.^15^ After confirming their structural identity as well as purity (>98%) by means of HPLC/UV, LC-time of flight-MS (LC-TOF-MS), and ^1^H NMR spectroscopy, the individual antioxidants were used as analytical standards for the quantitation by HPLC-MS/MS.

#### Synthesis of 4-Methoxyphenyl Glucoside (**45**)

The synthesis was performed based on a literature protocol with slight adaptions.^24^ Silver(I) carbonate (7 mmol) and 1,1,4,7,10,10-hexamethyltriethylentetramine (HMTTA, 3 mmol) were suspended in dry acetonitrile (20 mL) and stirred in the dark for 1 h. After adding 4-methoxyhydroquinone (2 mmol) and acetobromo-α-d-glucose (3 mmol) and stirring for further 30 min, the mixture was filtered (Schleicher & Schuell filter, 12 cm) and separated from solvent in vacuum at 40 °C. The raw synthesis was cleaned up on a Sepacore system (Büchi, Flawil, Switzerland) consisting of two C-605 pumps, a C-620 control unit, a C-660 fraction collector, and a C-635 UV detector. The separation was performed on a 150 mm × 50 mm i.d. 80 g Silica Flash Column (Kinesis, Cambridgeshire, U.K.). Operating with a flow rate of 40 mL/min, the solvent system consisted of *n*-hexane (A) and ethyl acetate (B), increasing the ratio from 20% B up to 50% B in 30 min and acquiring the absorption at a wavelength of 288 nm. The purified synthesis was separated from the solvent *via* rotary evaporation and dissolved in acetonitrile (10 mL) before deprotection with sodium hydroxide (1 M, 10 mL) dissolved in methanol/water (50/50, v/v). After 5 min, the mixture was neutralized with hydrochloric acid and separated by preparative HPLC on a 250 mm × 21.2 mm i.d., 5 μm, Luna Phenyl-Hexyl column (Phenomenex, Aschaffenburg, Germany) with a flow rate of 21 mL/min. Using a solvent system consisting of 0.1% formic acid in water (solvent A) and acetonitrile (solvent B), chromatography was performed by eluting with 20% B within 7 min, acquiring the effluent at a wavelength of 280 nm. Afterward, purified 4-methoxyphenyl glucoside was separated from the solvent in vacuum at 40 °C, followed by lyophilization.

#### Synthesis of 4-(2-Formylpyrrol-1-yl)butyric Acid (**37**) and 4-[2-Formyl-5-(hydroxymethyl)pyrrol-1-yl]butyric Acid (**38**)

The synthesis followed a literature protocol with modifications,^25^ obtaining **37** in a reaction with ribose as sugar and **38** using maltose. The respective sugar (20 mmol) and γ-aminobutyric acid (20 mmol) were dissolved in phosphate buffer (10 mL, 100 mM, pH 5.0) and heated for 6 h at 85 °C. The mixture was diluted with water (80 mL) and extracted with ethyl acetate (3 × 80 mL), before separating the combined organic extracts from solvent in vacuum at 40 °C. After dissolution in water, the compounds were purified by preparative HPLC, injecting onto a 250 mm × 21.2 mm i.d., 5 μm, Luna Phenyl-Hexyl column (Phenomenex, Aschaffenburg, Germany) with a flow rate of 21 mL/min using a 2 mL sample loop. Using a binary gradient of 0.1% formic acid in water (v/v) as solvent A and acetonitrile as solvent B, chromatography was performed with the following gradient for purification of **37**: 0 min/27% B, 7 min/27% B, 8 min/70% B, 11 min/70% B, 12 min/27% B, and 15 min/27% B. For **38**, a gradient was used as follows: 0 min/20% B, 6 min/20% B, 7 min/60% B, 10 min/60% B, 11 min/20% B, and 14 min/20% B. The effluent was acquired at a wavelength of 280 nm, and the collected compounds were separated from solvent in vacuum at 40 °C, followed by lyophilization.

#### Isomerization of 3-, 4-, and 5-Feruloylquinic Acid (**14**–**16**)

The isomerization was carried out in accordance with a literature protocol for chlorogenic acids.^26^ Separation and purification were performed by preparative HPLC using the same column and flow rate as those described above. Applying a binary gradient of 0.1% aqueous formic acid (v/v) as solvent A and acetonitrile as solvent B, chromatography was performed by keeping 15% B for 20 min. Monitoring the effluent at a wavelength of 280 nm, **14**–**16** were individually collected in several runs, combining the corresponding fractions. The fractions were separated from the solvent in vacuum at 40 °C, followed by lyophilization.

#### Isolation of *cis*-Isohumulone (**51a**) and Hydroxyl-*cis*-alloisohumulone (**53a**)

The iso-α-acids (**50a**–**c**, **51a**–**c**) were isolated using a commercial iso-α-acid extract (Hallertauer Hopfenveredelungsgesellschaft mbH, Mainburg, Germany), following a literature protocol with slight modifications.^10^ The iso-α-acid extract (10 g) was suspended in water (200 mL), and after adjusting the pH to 2.0 with formic acid extracted with ethyl acetate (2 × 200 mL), the combined organic extracts were separated from the solvent in vacuum at 40 °C and dissolved in a mixture of acetonitrile/water (70/30, v/v) before being injected onto a 250 mm × 21.2 mm inner diameter, 5 μm, HyperClone ODS column (Phenomenex, Aschaffenburg, Germany) at a flow rate of 21 mL/min. Monitoring the effluent at a wavelength of 272 nm, **50a**–**c** and **51a**–**c** were eluted with a mixture of acetonitrile/water (70/30, v/v) with 1% formic acid.

To obtain the hydroxyl-alloisohumulones (**52a**–**c**, **53a**–**c**), the iso-α-acid mixture was stored under oxygen atmosphere in the dark for 4 weeks. The raw product was dissolved in acetonitrile/water (50/50, v/v) and purified *via* the Sepacore system mentioned above. The separation was performed on a 460 mm × 16 mm i.d. glass column (Büchi, Flawil, Switzerland) filled with 25–40 μm LiChroprep RP18 material (Merck KGaA, Darmstadt, Germany). Operating with a flow rate of 30 mL/min, the solvent system consisted of aqueous formic acid (0.1%, A) and methanolic formic acid (0.1%, B), and the following gradient was used: 0 min/50% B, 15 min/80% B, 20 min/80% B, 25 min/100% B, and 40 min/100% B. Prior to the next injection, the column was flushed to 50% B for 5 min and kept for 10 min. The absorption at a wavelength of 234 nm was acquired with Sepacore Control Chromatography Software, version 1.0 (Büchi). The obtained mixture of congeners was further separated by preparative HPLC, injecting onto a 250 mm × 21.2 mm i.d., 5 μm, Hyperclone ODS column (Phenomenex, Aschaffenburg, Germany) with a flow rate of 21 mL/min. Using a binary gradient of 1% formic acid in water (A) and acetonitrile (B), the following chromatographic method was used: 0 min/50% B, 9 min/50% B, 12 min/100% B, 15 min/100% B, 16 min/50% B, 20 min/50% B. Based on the effluent at 234 nm, **52a**–**c** and **53a**–**c** were collected, separated from solvent in vacuum, and freeze-dried for 48 h.

### Sample Preparation

The beer samples were, after being degassed for 10 min upon ultrasonication and membrane filtration (Sartorius RC15 syringe filters, 0.45 μm), directly investigated by means of HPLC-MS/MS.

### High-Performance Liquid Chromatography–Triple Quadrupole Mass Spectrometry (HPLC-MS/MS)

For HPLC-MS/MS analysis, a Dionex UltiMate 3000 HPLC-system (Dionex, Idstein, Germany) was applied, consisting of a binary pump system (HPG-3400SD), a degasser (SRD-3400), an autosampler (WPS-3000TSL) set at 10 °C, and a column compartment (TCC-3000SD) set at 40 °C, using DC MS Link software version 2.8.0.2633 (Dionex) for HPLC instrument control. The HPLC was connected to an API 4000 QTrap triple quadrupole mass spectrometer (AB Sciex, Darmstadt, Germany) and an API 3200 QTrap triple quadrupole mass spectrometer (AB Sciex), respectively, operating in multiple reaction monitoring (MRM) mode using Analyst 1.5 software (AB Sciex) for data acquisition and instrument control. For tuning the mass spectrometer, methanol/water solutions (70/30, v/v) of each analyte were introduced by means of flow injection using a syringe pump (10 μL/min) using compound optimization tool of Analyst 1.5 software (AB Sciex).

### Quantitative Analysis of Phenolics

The analytical method was built on a published protocol,^27^ performed on the above-mentioned API 4000 QTrap triple quadrupole mass spectrometer system in negative electrospray ionization (ESI^–^) mode. Thereby, the ion spray voltage was set at −4500 V and nitrogen served as curtain gas (30 psi), nebulizer gas (55 psi), and turbo gas (45 psi, 425 °C). The characteristic mass transitions of the pseudo-molecular ions [M – H]^−^ into specific product ions were induced by collision-induced dissociation (CID) for 20 ms and are summarized in the Supporting Information (Table S2).

#### Internal Standard (IS)

Prior to the quantitative analysis, a stock solution of the internal standards was prepared, containing the phenylglucoside **45** (695.0 μmol/L) to quantify **17**–**18**, **19a**–**c**, **39**–**41**, benzoic acid derivative **46** (105.0 μmol/L) as IS for **1**–**3**, cinnamic acid derivative **47** (135.5 μmol/L) as IS for **4**–**9**, **11**–**16**, and **37**–**38**, flavanonol **48** (49.0 μmol/L) as IS for **4**–**9**, **11**–**16**, and **37**–**38**, and flavone **49** (21.5 μmol/L) as IS for **24**–**31**. The solution was kept at −20 °C until further use.

#### Analysis of Phenolics

Aliquots of each sample and standard solution (1 mL) were spiked with the IS (20 μL) and then investigated in triplicate by HPLC-MS/MS. Aliquots (5 μL) were injected onto an analytical Luna C18 column (150 mm × 2.0 mm i.d., 5 μm, Phenomenex, Aschaffenburg, Germany) at a flow rate of 0.3 mL/min, using the following solvent gradient with aqueous ammonium acetate buffer (5 mM, pH 5.0) as solvent A and ammonium acetate buffer (5 mM, pH 5.0) in acetonitrile/water (95/5, v/v) as solvent B: 0 min/0% B, 1 min/0% B, 3 min/20% B, 9 min/35% B, 10 min/100% B, 13 min/100% B, 15 min/0% B, 20 min/0% B.

#### Calibration Curve and Linear Range

After HPLC-MS/MS analysis, calibration curves were calculated by plotting the peak area ratios of analyte to internal standard against the concentration ratios of each analyte to the IS using linear regression and forcing the functions through zero to avoid negative or exaggerated results at the low end of the concentration ranges. The resulting correlation coefficients of all of the reference compounds were >0.99 (Table 1). **19b** and **19c** were quantified using calibration of the structurally related **19a**, assuming a response factor of 1.

**Table 1:** Calibration and Validation Data of the Quantitation of Antioxidants

	calibration				recovery		
no.^a^	calibration range [μmol/L]	function	*R*^2^	precision (*n* = 5) [%]	spiking range [μmol/L]	recovery rate [%]	*R*^2^
**1**	0.39–42.29	*y* = 0.642*x*	1.000	7.38			
**2**	0.44–28.00	*y* = 0.550*x*	0.999	13.53	0.70–5.60	111.0	0.936
**3**	0.14–24.87	*y* = 0.300*x*	0.998	8.97			
**4**	0.09–27.59	*y* = 1.468*x*	0.998	3.14			
**5**	0.03–29.02	*y* = 1.244*x*	0.998	12.68			
**6**	0.06–61.17	*y* = 0.847*x*	0.999	6.49	1.53–12.23	110.3	0.999
**7**	0.01–18.51	*y* = 0.487*x*	1.000	7.10			
**8**	0.25–185.24	*y* = 0.123*x*	0.999	8.70	4.63–37.05	108.9	0.989
**9**	0.13–13.72	*y* = 0.276*x*	1.000	6.92			
**10**	0.38–51.09	*y* = 0.152*x*	0.998	2.54	1.28–10.22	109.7	0.997
**11**	0.01–3.93	*y* = 0.832*x*	1.000	8.17			
**12**	0.03–18.64	*y* = 0.449*x*	0.998	6.07			
**13**	0.20–7.96	*y* = 0.126*x*	0.999	8.89			
**14**	0.02–12.63	*y* = 0.290*x*	1.000	16.00			
**15**	0.01–15.42	*y* = 0.653*x*	1.000	12.20	0.39–3.08	97.2	0.994
**16**	0.03–13.71	*y* = 0.232*x*	0.999	6.48			
**17**	0.11–20.76	*y* = 0.461*x*	1.000	7.92	0.52–4.15	100.4	0.998
**18**	0.43–72.11	*y* = 0.255*x*	0.999	4.10	1.80–14.42	104.9	0.991
**19a**	0.02–41.86	*y* = 12.485*x*	0.998	4.14	1.05–8.37	95.9	0.998
**19c**	0.02–41.86	*y* = 12.485*x*	0.998	7.64			
**19b**	0.02–41.86	*y* = 12.485*x*	0.998	6.63			
**20**	0.37–46.78	*y* = 0.568*x*	0.999	7.92			
**21**	0.37–17.91	*y* = 0.568*x*	0.999	12.34	0.45–3.58	108.2	0.994
**22**	0.34–25.06	*y* = 0.049*x*	0.997	9.87	0.63–5.01	115.6	0.992
**23**	0.65–32.80	*y* = 0.039*x*	1.000	4.98	0.82–6.56	111.0	0.997
**24**	0.01–22.30	*y* = 10.293*x*	0.996	6.49			
**25**	0.02–22.29	*y* = 9.498*x*	0.998	5.04			
**26**	0.00–25.23	*y* = 12.018*x*	0.998	8.13			
**27**	0.02–14.97	*y* = 1.818*x*	0.996	7.93			
**28**	0.02–13.26	y = 1.935*x*	0.999	2.57			
**29**	0.01–28.93	*y* = 2.118*x*	0.995	3.22			
**30**	0.03–14.48	*y* = 0.741*x*	0.999	7.84			
**31**	0.02–7.89	*y* = 2.058*x*	0.995	5.97	0.20–1.58	86.2	0.996
**32**	2.87–425.32	*y* = 0.134*x*	1.000	3.03	10.63–85.06	94.6	0.995
**33**	5.77–475.17	*y* = 0.371*x*	0.999	2.80	11.88–95.03	87.6	0.991
**34**	0.41–39.10	*y* = 0.075*x*	0.999	2.36	0.98–7.82	105.5	0.999
**35**	0.17–42.60	*y* = 0.126*x*	0.999	6.61	1.07–8.52	108.1	1.000
**36**	0.09–5.49	*y* = 0.161*x*	0.999	12.34	0.14–1.10	104.6	0.998
**37**	0.08–67.27	*y* = 0.458*x*	0.995	3.26			
**38**	0.02–36.93	*y* = 1.082*x*	0.995	7.62			
**39**	0.07–5.27	*y* = 0.769*x*	0.999	11.88			
**40**	0.16–16.38	*y* = 0.461*x*	0.998	10.73			
**41**	0.17–11.74	*y* = 0.419*x*	1.000	4.36			
**42**	0.04–15.01	*y* = 0.206*x*	1.000	9.57	0.38–3.00	103.1	0.999
**43**	0.04–28.10	*y* = 0.279*x*	1.000	8.25	0.70–5.62	107.5	1.000
**44**	0.01–2.75	*y* = 0.825*x*	0.999	14.35	0.07–0.55	102.4	0.999

a Chemical structures are given in Figure 1.

### Quantitative Analysis of Amino Acids (32–33), Hordatines (34–36), and Hordatine Glucosides (42–44)

For the quantitative investigation, the API 3200 QTrap triple quadrupole mass spectrometer, operating in ESI^+^ mode, was used with an ion spray voltage of +5500 V and nitrogen as the curtain gas (35 psi), nebulizer gas (55 psi), and turbo gas (65 psi, 550 °C). The characteristic mass transitions of the pseudo-molecular ions ([M + H]^+^, and [M + 2H]^2+^ for **34**–**36** and **42**–**44**) into specific product ions were induced by collision-induced dissociation (CID) for 50 ms and are summarized in the Supporting Information (Table S3).

#### Internal and ECHO Standard

For quantitation of **32** and **33**, an internal standard solution was prepared, containing **32a** (578 μmol/L) and **33a** (492 μmol/L). Moreover, the ECHO technique was applied,^28^ using an ECHO standard solution containing **10** (5.11 μmol/L), **35** (8.52 μmol/L), and **43** (2.81 μmol/L) to perform quantitation of **10**, **34**–**36**, and **42**–**44**, respectively.

#### Analysis of Amino Acids (**32**–**33**), Hordatines (**34**–**36**), and Hordatine Glucosides (**42**–**44**)

Aliquots of each sample and standard solution (1 mL) were spiked with the internal standard (20 μL), and aliquots (5 μL) were measured in triplicate by HPLC-MS/MS. The analysis was carried out on an analytical Luna PFP column (150 mm × 2.0 mm i.d., 3 μm, Phenomenex, Aschaffenburg, Germany) at a flow rate of 0.2 mL/min, using the following solvent gradient with aqueous formic acid (1%) as solvent A and acetonitrile with 1% formic acid as solvent B: 0 min/0% B, 1 min/0% B, 11 min/45% B, 12 min/45% B, 14 min/100% B, 16 min/100% B, 18 min/0% B, 21 min/0% B. After 7 min, an aliquot of the ECHO standard solution (5 μL) was injected additionally onto the column.

#### Calibration Curve and Linear Range

After measurement by HPLC-MS/MS, calibration curves were calculated by plotting the peak area ratios of analyte to internal or ECHO standard against concentration ratios of each analyte to the internal or ECHO standard. Thereby, linear regression functions were used, and the curves were forced through zero, leading to correlation coefficients of >0.99 (Table 1).

### Validation of the Developed Quantitation Methods for Antioxidants

#### Limit of Detection (LOD)/Limit of Quantitation (LOQ)

The calculation of the LOD and LOQ was performed by integrating the noise in a beer sample over one usual peak width right before the analyte peak prior to calculation of a theoretical concentration using the calibration curve. The resulting value was multiplied by a factor of 3 or 10 to express the LOD or LOQ.

#### Precision

Five aliquots of the same standard mixture and beer sample were analyzed in the same batch on consecutive days. The precision of the developed method, expressed by the relative standard deviation (%) of the five replicates, is given in Table 1 for each analyte.

#### Recovery

After the concentrations of antioxidants in a beer sample (control) were determined, the sample was spiked with four different levels of the chosen antioxidants (Table 1). After sample workup as reported above and quantitation by means of HPLC-MS/MS, the recovery rate was calculated by plotting the measured concentration against the added concentration and is expressed as the slope of the regression line from a simple regression (Table 1).

### Spiking Experiments and Storage Trials

For every batch of the spiking experiments, the content of chosen antioxidants (Table 3) in pilsner beer (3 mL) was mixed as methanolic solutions, and the solvent was removed under a stream of nitrogen at room temperature. After degassing for 10 min upon ultrasonication, each beer (3 mL) was added before vortexing. The storage was carried out under argon at 40 °C without stirring. Samples were collected before starting the incubation and after 7, 14, and 21 days, respectively, membrane-filtered (Sartorius RC15 syringe filters, 0.45 μm) and kept at −20 °C until analyzing by LC-MS/MS.

### Quantitative Analysis of Oxidation Indicators

The analysis of **50a**–**c** to **53a**–**c** was based on a literature protocol with some modifications.^29^ Thereby, the above-mentioned API 4000 QTrap triple quadrupole mass spectrometer in the ESI^–^ mode was used. The ion spray voltage was set at −4500 V and nitrogen served as curtain gas (30 psi), nebulizer gas (50 psi), and turbo gas (60 psi, 625 °C). The characteristic mass transitions of the pseudo-molecular ions [M – H]^−^ into specific product ions were induced by collision-induced dissociation (CID) for 50 ms and are summarized in the Supporting Information (Table S4).

#### ECHO-Standard

Two ECHO standard solutions, with the first containing **53a** (2.48 μmol/L) and the second containing **51a** (35.3 μmol/L), were prepared for quantitation of **52a**–**c** to **53a**–**c**, and **50a**–**c** to **51a**–**c**, respectively.

#### Analysis of Oxidation Indicators

Aliquots of each sample (5 μL, each analyzed in triplicate) were measured by HPLC-MS/MS. The samples were injected into an Accucore Phenyl-Hexyl column (150 mm × 2.1 mm i.d., 2.6 μm, Phenomenex, Aschaffenburg, Germany) at a flow rate of 0.7 mL/min and using the following solvent gradient with aqueous formic acid (1%) as solvent A and acetonitrile with 1% formic acid as solvent B: 0 min/30% B, 10 min/60% B, 12 min/7% B, 13 min/100% B, 15 min/100% B, 16.5 min/30% B, 18.5 min/30% B. After 2.5 and 7.5 min, an aliquot of the first and second ECHO standard (5 μL, each) was injected additionally onto the column.

#### Calibration Curve and Linear Range

After data acquisition, calibration curves were calculated by plotting the peak area ratios of the analyte to ECHO standard against concentration ratios of each analyte to the ECHO standard. Linear regression functions were used, and the curves were forced through zero, leading to correlation coefficients of >0.99.

### Data Analysis

Data analysis was performed within the programming and visualization environment R (version 2.10.0).^30^ The heatmap was calculated using the heatmap.2 function of “gplots” package based on the raw concentration data (Tables S5 and S6) and the dendrogram was constructed by means of an agglomerative average linkage algorithm,^31^ whereas the distance between two clusters is defined as the average of distances between all pairs of objects and each pair is made up of one object from each group.

### Storage Trials under Oxygen Atmosphere

For investigation of oxygen-dependent aging reactions, two batches of beer (10 mL, each) were stirred in the dark under oxygen atmosphere for up to 28 days at room temperature. To ensure a constant oxygen atmosphere, the sample vial was connected *via* a capillary to a balloon filled with oxygen. Samples (1 mL) were collected after 2, 3, and 4 weeks, and the oxygen reservoir was refilled every week. Additionally, 50 mL was kept at −20 °C as a fresh beer control. After analysis by UPLC-TOF-MS, data were processed using Progenesis QI (Waters, Manchester, U.K.), as the *S*-plot was calculated after OPLS-DA with EZ-Info as software (Waters, Manchester, U.K.).

### UPLC/Time-of-Flight Mass Spectrometry (UPLC-TOF-MS)

Aliquots (2 μL) of the samples were injected into an Acquity UPLC core system (Waters, Manchester, U.K.), consisting of a binary solvent manager, a sample manager, and a column oven. The chromatographic separation was performed on a 150 mm × 2 mm i.d., 1.7 μm, BEH C18 column (Waters, Manchester, U.K.) at a flow rate of 0.4 mL/min and a temperature of 40 °C. Aqueous formic acid (0.1%, A), and acetonitrile (B) were used as solvents, and for purity investigations, the following gradient was used: 0 min/5% B, 3 min/100% B, and 4 min/100% B. For the storage trials, separation was performed as follows: 0 min/5% B, 10 min/100% B, and 11 min/100% B. High-resolution mass spectra were recorded on a Synapt G2-S HDMS (Waters, Manchester, U.K.) in negative and positive ESI resolution modes using −3.0 and +2.5 kV capillary voltage, respectively, 30 kV sampling cone, 4.0 kV extraction cone, 150 °C source temperature, 450 °C desolvation temperature, 30 L/h cone gas, and 850 L/h desolvation gas. The instrument was calibrated (*m*/*z* 50–1200) using a solution of sodium formate (0.5 mM) dissolved in 2-propanol/water (9/1, v/v). All data were lock mass corrected using leucine enkephaline as the reference (*m*/*z* 554.2615, [M – H]^−^ and *m*/*z* 556.2771, [M + H]^+^). Data acquisition and interpretation were performed using MassLynx (version 4.1) and the tool “elemental composition” as software.

### Estimation of the Antioxidant Activity *In Vitro*

The antioxidant capability of the purified compounds was measured by applying three *in vitro* assays that cover different mechanisms of antioxidants, namely, oxygen radical absorbance capacity (ORAC), hydrogen peroxide scavenging (HPS), and linoleic acid (LA) assay, following the previously described protocol.^14^ Thereby, the ORAC assay focuses mainly on the radical scavenging potential, which decelerates the degradation of fluorescein. The HPS assay covers the direct reduction of hydrogen peroxide and inhibition of peroxidase, besides the main mechanism of quenching the formed radicals instead of ABTS as a substrate. The LA assay finally covers the direct reduction of hydrogen peroxide and the iron(II) chelating potential of antioxidants and works with naturally relevant linoleic acid as substrate for generated radicals.

### Nuclear Magnetic Resonance (NMR) Spectroscopy

Purity investigations of isolated compounds were performed in accordance with a literature protocol.^32^^1^H and ^13^C NMR-spectra were recorded for synthesized compounds on a 400 MHz ultrashield Avance III spectrometer with a Broadband Observe BBFOplus probe head and a 500 MHz ultrashield plus Avance III spectrometer with a Triple Resonance Cryo Probe TCI probe head (Bruker, Rheinstetten, Germany), respectively. Using methanol-*d*_4_ and D_2_O as solvents, the chemical shifts were reported in parts per million relative to the solvent signal. Data processing was performed using XWin-NMR version 3.5 (Bruker, Rheinstetten, Germany) and Mestre-Nova 8 (Mestrelab Research, Santiago de Compostela, Spain) as software.

## Results and Discussion

To clarify the impact of very recently identified beer compounds in comparison to well-known contributors to the antioxidant activity of different beer types, an accurate and sensitive quantitation technique should be developed using HPLC-MS/MS. By using an easy and gentle sample workup, it should be verified, moreover, that the antioxidants occur naturally and were not a workup artifact. Thereby, well-known compounds, such as **20**–**23**,^12,33^ and **39**–**40**,^34,35^ were considered as well as recently reported structures (Figure 1).^14^ Regarding recently published antioxidant precursors as well as **33** as a precursor of **8**,^15,36^ their residual concentration in beer should be studied, too. As **24** and **25** were found in beer,^14,37^ associated **26** was also considered for quantitation, being reported in barley leaves,^38^ as well as **12**, known from beer and spent grain,^39,40^ while being similar to **13**. **17**, furthermore, was analyzed as a homologue of **18**, with **39** and **40** being characterized in barley as precursors.^15^

### Method Development for the LC-MS/MS Analysis of Antioxidants 1–44

**Figure 2:**
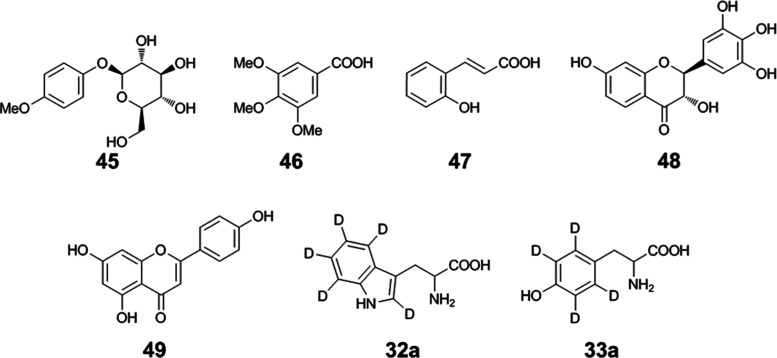
Chemical structures of compounds applied as internal standards: 4-methoxyphenyl glucoside (**45**), 3,4,5-trimethoxybenzoic acid (**46**), *o-*coumaric acid (**47**), dihydrorobinetin (**48**), apigenin (**49**), tryptophan-*d*_5_ (**32a**), and tyrosine-*d*_4_ (**33a**).

**Figure 3:**
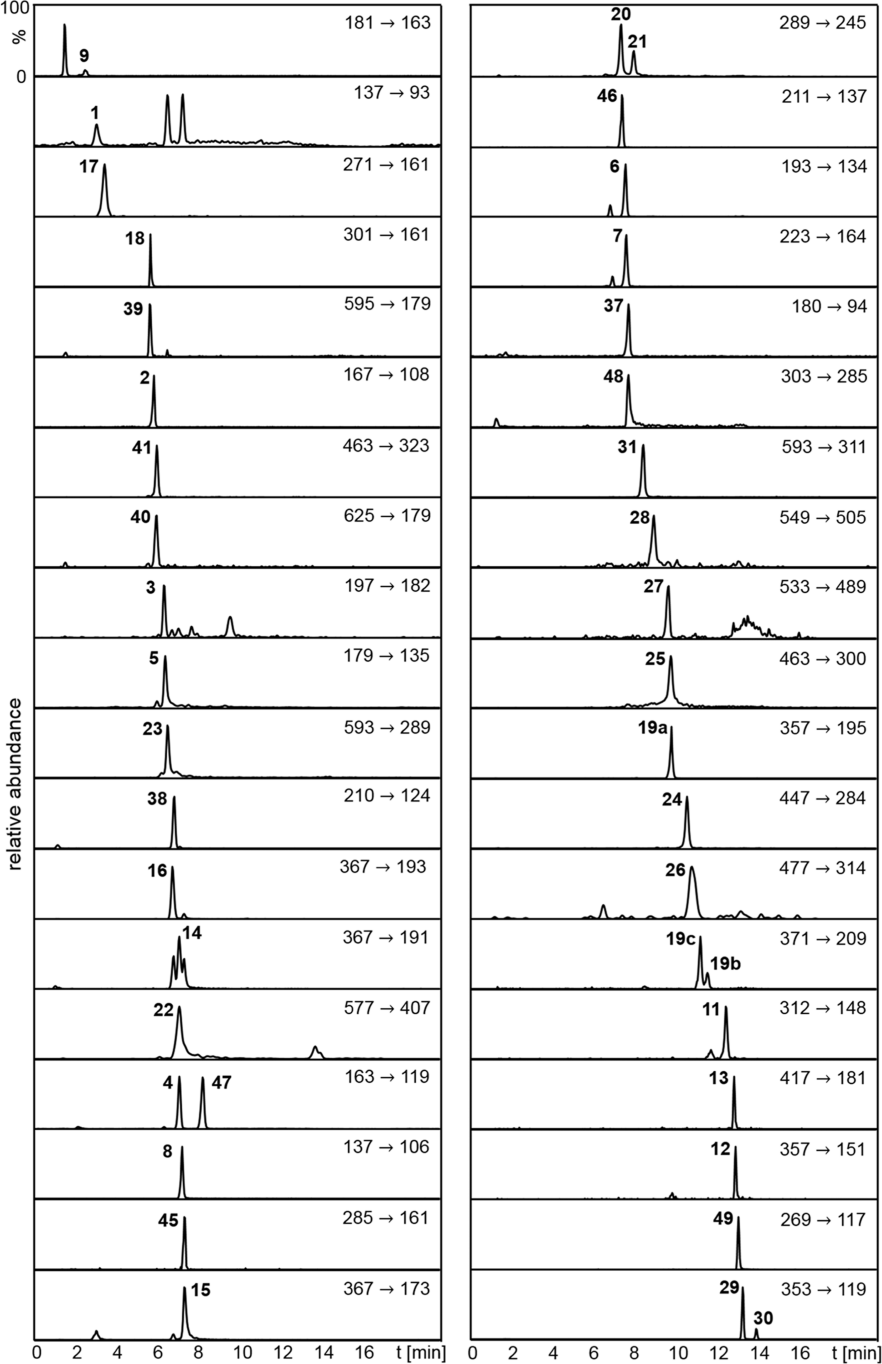
HPLC-MS/MS analysis of a beer sample showing the mass transition traces of the phenolic compounds and internal standards analyzed in ESI^–^ mode. Signal intensity of each mass transition is normalized and numbering of compounds refers to chemical structures given in Figures 1 and 2.

**Figure 4:**
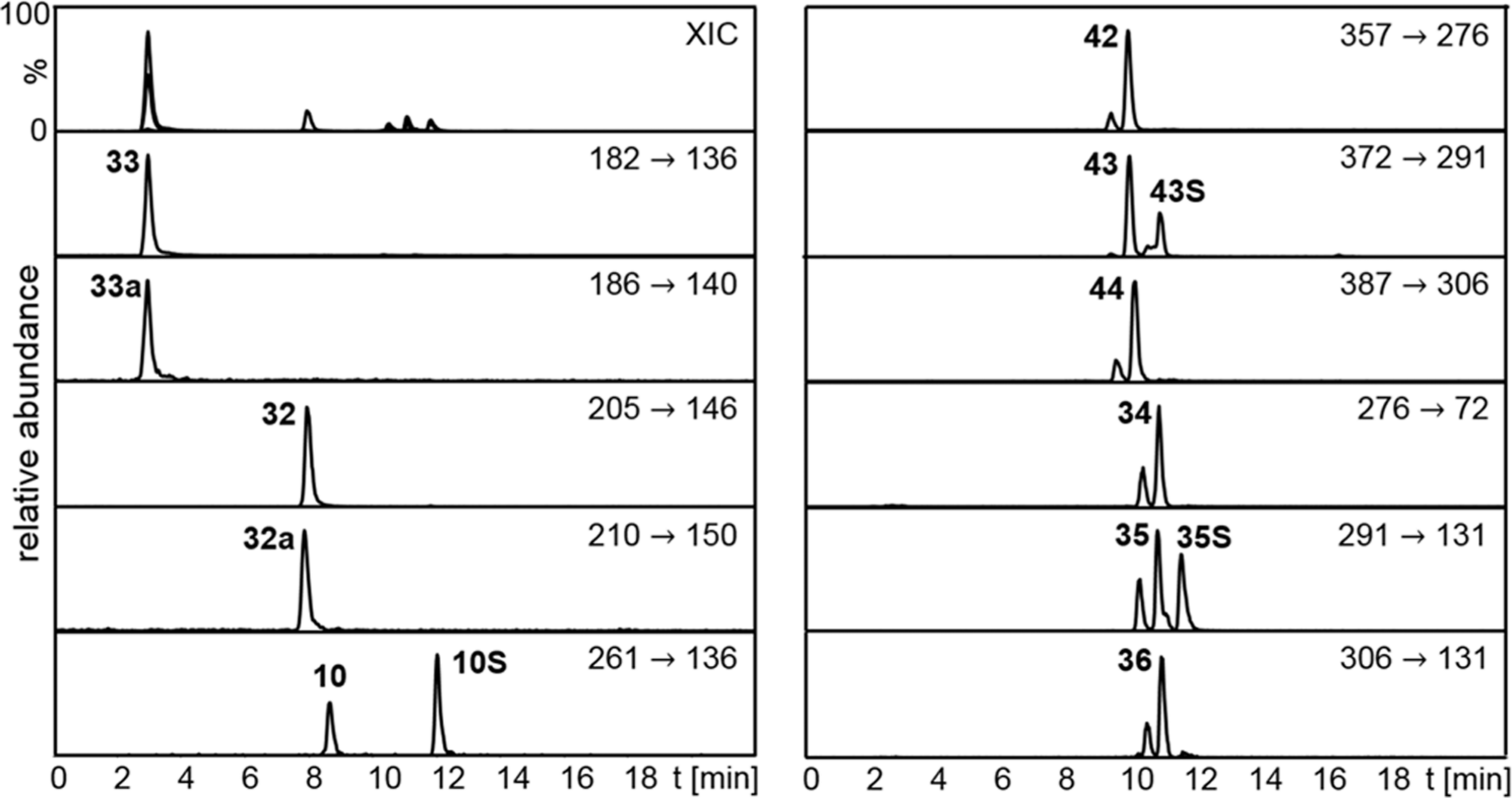
HPLC-MS/MS analysis of a beer sample showing the mass transition traces of the antioxidants and internal standards analyzed in ESI^+^ mode. Signal intensity of each mass transition is normalized and numbering of compounds refers to chemical structures given in Figures 1 and 2, as ECHO standards are marked with an “S”.

To reach the highest selectivity, MRM mode was used for quantitation, as successfully applied to red wine phenolics or beer bitter constituents.^27,29,37,41^ The MS/MS parameters were optimized to maximize the product ion intensity and increase the sensitivity of the method by infusing every single reference compound into the mass spectrometer using a syringe pump (Figure 2). Besides the most abundant mass transition used for quantitation (Figures 3 and 4), a second specific ion transition was selected for unambiguous identification of the target compound. Furthermore, the chromatographic separation ensured the distinction between analytes with similar mass transitions. Since **10**, **32**, and **33** revealed a higher sensitivity for the ions [M + H]^+^, and both **34**–**36** and **42**–**44** formed in the ion source predominantly the ions [M + 2H]^2+^, they were analyzed in ESI^+^ mode, being tuned on the most abundant mother ion (Figure 4). The other antioxidants were analyzed in negative ESI mode using the pseudo-molecular ion [M – H]^−^ (Figure 3). The quantitation was performed based on the quantitation of phenolics in red wine,^27,41^ applying 3,4,5-trimethoxybenzoic acid (**46**) as internal standard for **1**–**3**, *o-*coumaric acid (**47**) for **4**–**9**, **11**–**16**, and **37**–**38**, dihydrorobinetin (**48**) for **20**–**23**, and apigenin (**49**) for **24**–**31** (Figure 2). Additional synthesis of **45** enabled the quantitation of phenylglucosides **17**–**18**, **19a**–**c**, and **39**–**41**. **32** and **33** were analyzed using isotopes labeled **32a** and **33a** as standards, whereas no appropriate internal standard was available for **10**, **34**–**36**, and **42**–**44**. To overcome this challenge, the ECHO technique, as already used for the analysis of beer bitter constituents,^29,35^ was applied, utilizing **10**, **35**, and **43** as ECHO standards. In order to evaluate the robustness of the quantitation methods, accuracy experiments were carried out. The precision was investigated by analyzing a single sample in a fivefold injection spread over different days, revealing a relative standard deviation ranging from 2.36 to 16.00% (Table 1). Similarly, the reproducibility was expressed by the coefficient of variation of three independent sample preparations and revealed values 1.08 and 12.24%. In addition, the recovery rate was studied for the chosen antioxidants from the different compound classes. For this purpose, a beer sample was spiked with four different concentration levels of antioxidants before analyzing their total content. The levels were guided by the average natural beer concentration from a pretest, with a spiking range from 20 to 160% of the estimated amounts. Using an additional unspiked sample (control), as well, the spiked concentration was plotted against the measured concentration, with the recovery rate being expressed as the slope of the regression line after simple regression. Thereby, reliable analytical results could be obtained with recoveries ranging from 86.2 to 115.6%.

### Concentrations of Antioxidants in Beer Samples

Intending to get an overview of the naturally occurring content of the antioxidants, 23 commercial beer samples without antioxidant additives were investigated using the developed HPLC-MS/MS method (Table S1, Supporting Information). Since many antioxidants were shown to be cereal-derived and partially being released during fermentation,^15^ a range of beer types was covered with focus on different combinations of utilized malt and type of fermentation. Besides four pilsner (I–IV) and five pale lager (V–IX) beers (bottom-fermented, using only pale barley malt, using hop and/or hop extracts), two dark lager beers (X–XI) were investigated, introducing a portion of special malt types to achieve the dark color. Utilizing a mixture of malt from wheat and barley, four pale (XII–XV) and two dark (XVI–XVII) top-fermented wheat-type beer samples were analyzed, too. To also consider some special-type beers, a strong beer with higher original wort (XVIII), a Munich dark (XIX), and a stout (XX), containing special dark roasted malt types, were analyzed along with two pale ale (XXI–XXII) (top-fermented, using only barley malt) and one India pale ale beer (XXIII) being characterized by a higher and late hop dosage.

**Figure 5:**
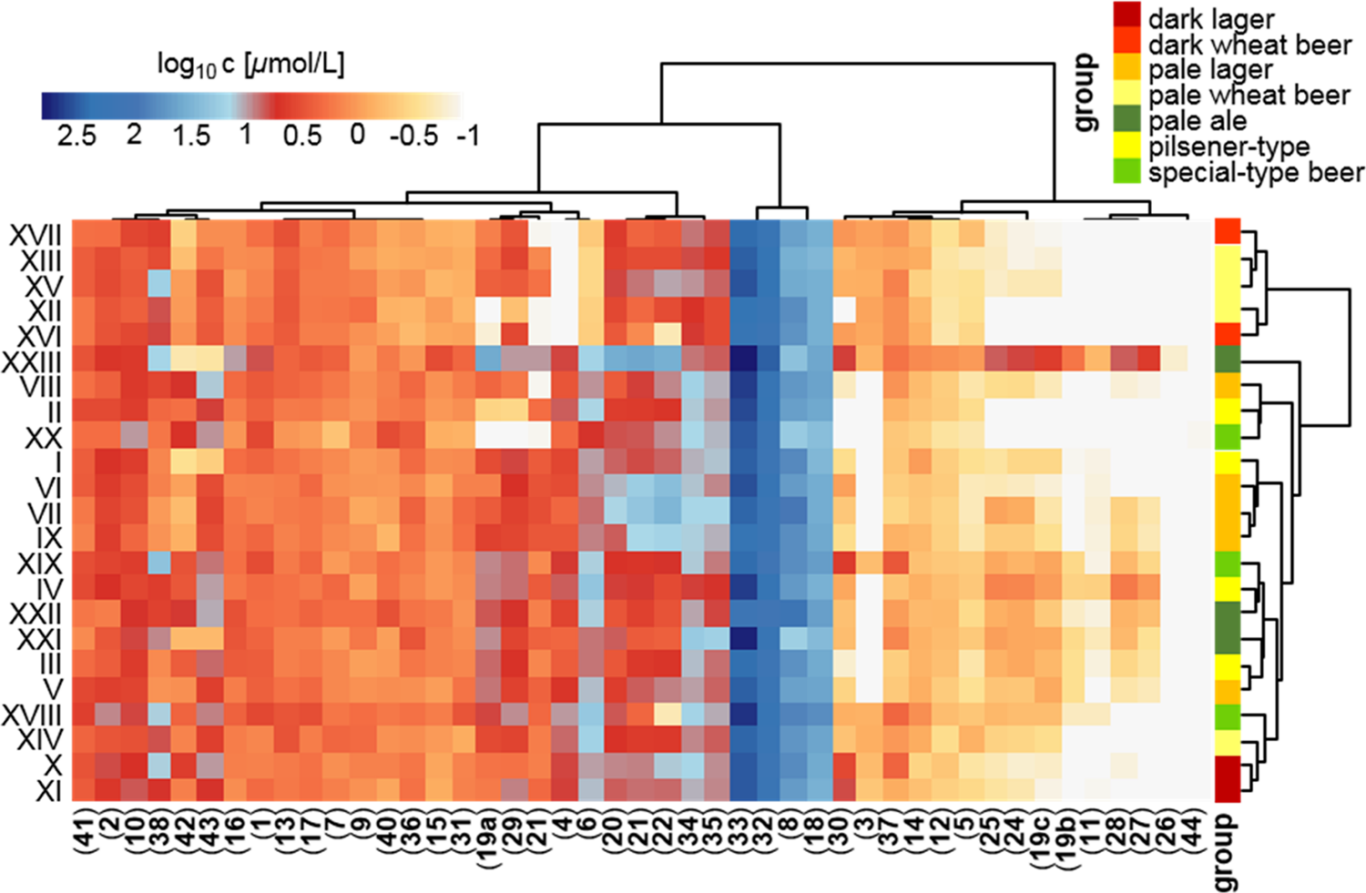
Hierarchical cluster analysis of the concentrations of antioxidants and antioxidant precursors in commercial beer samples scaled logarithmically. Numbering of compounds refers to chemical structures given in Figure 1.

The determination of the antioxidants’ content revealed a wide field of measured values, ranging from an average concentration of 0.01 μmol/L for **26** to about a 30,000 times higher concentration for **33** with nearly 300 μmol/L (Table 2). Therefore, the data was log-transformed and plotted in the heatmap (Figure 5), highlighting a cluster of quantitatively dominating constituents common in all investigated beers. Besides the amino acids **33** and **32** with 300 and 160 μmol/L, also **8** and **18** revealed a high content of 48 and 33 μmol/L, respectively. Especially the importance of **18** was remarkable, having been published in beer only recently.^14^ The homologue **17**, however, occurred with a lower concentration of 1.68 μmol/L on average. In the case of **8**, remarkable quantitative differences between samples were observed, with levels ranging from 15.1 to 126 μmol/L. Since all of the three beer samples with significantly lower levels are top-fermented stouts and ales, this might be linked to varying yeast types used for fermentation, manifesting a lower Ehrlich degradation activity to generate **8**.^36^

**Table 2:** Mean Concentrations, Concentration Range, Antioxidant Activity, and Resulting Activity Values of Antioxidants in Beer

	concentration [μmol/L]		antioxidant activity [μmol TE/μmol]			activity value^b^ [μmol TE/L beer]		
no.^a^	average	natural variation	ORAC-assay^c^	HPS-assay^d^	LA-assay^d^	ORAC	HPS	LA
**1**	2.11	1.18–5.73	4.92 ± 0.40^e^	1.08 ± 0.08^e^	<0.01 ± 0.00^e^	10.37	2.27	0.00
**2**	3.39	1.41–7.69	3.39 ± 0.12^e^	1.26 ± 0.14^e^	0.41 ± 0.03^e^	11.49	4.27	1.38
**3**	0.50	>0.04–0.83	1.16 ± 0.18^e^	1.49 ± 0.13^e^	1.27 ± 0.13^e^	0.58	0.74	0.63
**4**	3.29	>0.03–8.55	3.81 ± 0.38^e^	0.95 ± 0.09^e^	0.15 ± 0.02^e^	12.56	3.14	0.49
**5**	0.33	0.18–0.92	4.17 ± 0.12^e^	1.17 ± 0.09^e^	2.75 ± 0.59^e^	1.36	0.38	0.90
**6**	8.16	0.31–18.3	5.48 ± 0.13^e^	1.10 ± 0.05^e^	0.86 ± 0.12^e^	44.72	8.97	7.02
**7**	1.37	0.46–2.11	2.10 ± 0.22^e^	1.34 ± 0.08^e^	1.13 ± 0.18^e^	2.88	1.84	1.55
**8**	48.5	15.1–126	1.33 ± 0.18^e^	0.78 ± 0.05^e^	0.04 ± 0.00^e^	64.36	37.87	1.95
**9**	1.20	0.76–1.88	0.80 ± 0.08^e^	1.79 ± 0.23^e^	<0.01 ± 0.00^e^	0.95	2.15	0.00
**10**	3.87	1.98–8.68	1.12 ± 0.08^e^	0.50 ± 0.04^e^	<0.01 ± 0.00^e^	4.34	1.95	0.00
**11**	0.13	0.02–0.58	4.27 ± 0.45^e^	0.68 ± 0.03^e^	0.50 ± 0.02^e^	0.55	0.09	0.06
**12**	0.40	0.21–1.06	5.15 ± 0.21	1.51 ± 0.18	2.32 ± 0.35	2.05	0.60	0.93
**13**	1.84	0.98–2.81	2.28 ± 0.04^e^	1.21 ± 0.10^e^	0.80 ± 0.05^e^	4.21	2.23	1.47
**14**	0.61	0.33–1.18	1.69 ± 0.21^e^	0.85 ± 0.11^e^	1.08 ± 0.26^e^	1.02	0.51	0.66
**15**	1.04	0.60–2.97	1.63 ± 0.30^e^	0.83 ± 0.13^e^	1.37 ± 0.18^e^	1.70	0.86	1.43
**16**	1.88	0.86–8.78	1.87 ± 0.29^e^	0.83 ± 0.14^e^	1.13 ± 0.24^e^	3.51	1.56	2.13
**17**	1.68	0.90–2.89	3.61 ± 0.16	1.40 ± 0.13	0.39 ± 0.05	6.06	2.35	0.65
**18**	32.9	22.3–55.2	2.62 ± 0.14^e^	0.98 ± 0.16^e^	1.77 ± 0.23^e^	86.06	32.06	58.18
**19a**	4.55	<0.005–32.5	2.16 ± 0.03^e^	0.63 ± 0.08^e^	0.55 ± 0.07^e^	9.83	2.88	2.48
**19c**	0.18	<0.005–1.58	2.23 ± 0.10^e^	0.45 ± 0.08^e^	0.83 ± 0.08^e^	0.40	0.08	0.15
**19b**	0.46	<0.005–3.79	2.06 ± 0.11^e^	0.54 ± 0.08^e^	0.74 ± 0.07^e^	0.94	0.25	0.34
**20**	6.29	2.33–30.9	11.16 ± 0.33	2.67 ± 0.44	3.64 ± 0.36	70.23	16.82	22.92
**21**	1.99	>0.11–8.63	10.02 ± 0.87	3.84 ± 0.46	4.60 ± 0.53	19.97	7.66	9.17
**22**	7.11	1.15–35.7	12.66 ± 1.77	4.69 ± 0.53	11.92 ± 1.95	90.05	33.35	84.83
**23**	8.06	>0.19–30.3	10.04 ± 0.23	5.68 ± 0.67	9.81 ± 1.18	80.92	45.72	79.01
**24**	0.59	>0.002–5.40	4.75 ± 0.13^e^	1.59 ± 0.21^e^	0.51 ± 0.06^e^	2.79	0.93	0.30
**25**	0.63	>0.01–6.04	3.94 ± 0.20^e^	2.09 ± 0.34^e^	1.81 ± 0.26^e^	2.48	1.32	1.14
**26**	0.02	>0.001–0.15	4.13 ± 0.31	1.02 ± 0.12	1.06 ± 0.18	0.07	0.02	0.02
**27**	0.32	<0.01–3.94	4.45 ± 0.34^e^	1.95 ± 0.31^e^	0.91 ± 0.17^e^	1.41	0.62	0.29
**28**	0.49	<0.01–6.18	6.61 ± 0.45^e^	1.74 ± 0.29^e^	2.97 ± 0.46^e^	3.23	0.85	1.45
**29**	3.90	0.05–8.42	2.52 ± 0.08	0.45 ± 0.04	0.16 ± 0.02	9.84	1.75	0.63
**30**	1.14	>0.01–5.41	1.11 ± 0.05	0.14 ± 0.01	0.13 ± 0.02	1.26	0.16	0.14
**31**	1.22	0.60–2.59	12.92 ± 0.19^e^	2.02 ± 0.25^e^	1.17 ± 0.18^e^	15.71	2.46	1.42
**32**	161	104–270	2.05 ± 0.36^e^	0.19 ± 0.04^e^	<0.01 ± 0.00^e^	329.57	31.09	0.00
**33**	298	128–576	0.98 ± 0.13	0.15 ± 0.05	<0.01 ± 0.00	292.99	44.26	0.00
**34**	9.83	4.50–16.8	10.01 ± 0.97^e^	1.97 ± 0.44^e^	1.05 ± 0.13^e^	98.47	19.41	10.31
**35**	7.59	3.11–14.9	12.58 ± 0.98^e^	3.02 ± 0.53^e^	3.19 ± 0.32^e^	95.52	22.90	24.21
**36**	1.44	0.56–2.61	17.50 ± 0.77^e^	4.10 ± 1.01^e^	6.14 ± 1.28^e^	25.18	5.90	8.83
**37**	0.97	0.32–2.49	<0.01 ± 0.00^e^	<0.01 ± 0.00^e^	0.13 ± 0.01^e^	0.00	0.00	0.12
**38**	5.55	0.93–19.7	<0.01 ± 0.00^e^	<0.01 ± 0.00^e^	0.12 ± 0.01^e^	0.00	0.00	0.68
**39**	0.04	<0.02–0.11	2.95 ± 0.25^f^	1.78 ± 0.13^f^	0.89 ± 0.1^3^^f^	0.13	0.08	0.04
**40**	1.23	0.60–2.99	2.19 ± 0.22^f^	0.71 ± 0.05^f^	1.95 ± 0.18^f^	2.68	0.87	2.39
**41**	2.21	1.16–3.63	2.50 ± 0.13^f^	0.72 ± 0.12^f^	1.81 ± 0.20^f^	5.52	1.59	4.00
**42**	1.73	0.20–4.92	1.94 ± 0.16^f^	0.59 ± 0.05^f^	1.04 ± 0.22^f^	3.35	1.02	1.80
**43**	4.58	0.22–11.3	3.40 ± 0.26^f^	1.17 ± 0.19^f^	1.31 ± 0.04^f^	15.57	5.34	5.99
**44**	0.03	>0.002–0.11	4.76 ± 0.24^f^	0.46 ± 0.20^f^	1.01 ± 0.12^f^	0.12	0.01	0.03

a Chemical structures are given in Figure 1.

b Defined as the average concentration multiplied with the antioxidant activity.

c Errors express standard deviation of four replicates.

d Errors express the confidence interval (α = 5%) of each three replicates.

e Data taken from ref (14).^14^

f Data taken from ref (15).^15^

Further quantitatively important compounds, including **34**, **35**, and **7**, connected to **23**, **22**, and **20**, were combined in another cluster (Figure 5), which comprises different mainly malt-derived components.^15^ Especially for **34** and **35**, having not been quantified in beer previously, remarkably high contents were measured with means of 9.83 and 7.59 μmol/L, respectively, revealing their importance in beer. They were in the same range as **7** (8.16 μmol/L), **23** (8.06 μmol/L), and **22** (7.11 μmol/L), which fitted well to published data.^16,42^ Thereby, the concentration ratio among the hordatines (**34**–**36**) and hordatine glucosides (**42**–**44**) varied significantly, as also observed in malt,^15^ highlighting the modulating potential that seems to be linked to the malting and mashing conditions. Although **34** was the quantitatively dominating aglycone, or in some samples at an equal level as **35**, **43** was the predominant glucoside in nearly all investigated samples, occurring at about three times higher levels than **42**, except in beers with low hordatine glucoside levels of <0.6 μmol/L (beer I, XXI and XXIII). For the other group of antioxidant precursors, **39**–**41**, a narrower natural range was determined. The concentration of **39**, thereby, was below the level of quantitation, although it was detectable in all samples. Nevertheless, e.g., the content of **41**, ranging from 1.16 to 3.63 μmol/L, was more consistent than for **43**, ranging from 0.22 to 11.33 μmol/L, indicating a quite reproducible degradation as reported during mashing and fermentation.^15^ Enormous variations were also observed for hop-derived antioxidants, such as **29**, ranging from 0.05 to 8.42 μmol/L, and **19a**, which was not even detectable in the investigated stout (XX), whereas a content of 32.55 μmol/L was determined for the India pale ale sample (XXIII), being far above the mean value of 4.55 μmol/L. This is driven by the amount and type of hopping, reflecting well the reported characteristics of different hop products, too.^37^

The hierarchical cluster analysis of the different beer samples revealed, moreover, a similar pattern for nearly all investigated wheat-type beer samples, particularly differing in the contents of **6** and **4** (Figure 5). Although average concentrations of 9.67 and 3.61 μmol/L were recorded for pilsner-type and pale lager beers, amounts of <0.5 μmol/L were measured for **6** in all wheat-type beers except XIV, as **4** even could not be detected. This cannot be exclusively explained by wheat malt characteristics, since wheat-type beers contain a portion of malt from barley as well, which can be confirmed by the amount of barley-specific components **34**–**36**. Instead, it seems that the specific yeast strains of top-fermented wheat-type beer and characteristics of their metabolism also have an impact.

Despite the above-mentioned differences in the antioxidant profile of individual beer samples describing the natural range, substantiated average concentrations could be derived to allow for a generic judgment. Thereby, the average concentrations in pale lager and pilsner-type beer (I–IX) were found to be close to the average value of all investigated beer samples (I–XXIII), although the natural range is much larger for all beer samples. Exemplarily, for **8**, 48.5 μmol/L on average for all investigated beer samples (I–XXIII) and 51.1 μmol/L on average for pale lager and pilsner-type samples (I–IX) were comparable, being at the same time well in line with literature values.^43^**43** with 4.58 on average compared to 5.02 μmol/L and **29** with 3.90 compared to 3.72 μmol/L showed the same tendency, as well as **18** with 32.9 and **34** with 9.83 μmol/L on average for all samples, compared to 32.7 and 10.6 μmol/L in pale lager and pilsner-type beers. Just for **38**, the concentration in pale lager and pilsner-type beers (I–IX) of 1.75 μmol/L was below the overall average content of 5.55 μmol/L, which can be explained by the lower torrefying degree of utilized pale malt, fitting to the findings for malt samples.^15^

Summarizing, the qualitative composition of antioxidants was reproducible for all samples, except for some hop-derived constituents. The quantitative profile, however, exhibited notable variabilities between samples, connected with the brewing recipe, although substantiated average concentrations could be derived.

### Dose-Activity Considerations of Antioxidants in Beer

To reveal the antioxidants with the highest impact on the antioxidant activity of beer, quantitative data was combined with the antioxidants’ activity for purified compounds (Table 2). A ranking approach for the antioxidants was applied similar to studies on sensometabolites, calculating the taste activity value (TAV), or dose-overthreshold factor (DoT), respectively.^44,45^ Thereby, multiplying the average concentration in beer and the *in vitro* antioxidant activity of a single compound, applying the oxygen radical absorbance capacity (ORAC) assay, the hydrogen peroxide scavenging (HPS) assay and the linoleic acid (LA) assay, led to the antioxidant activity value, expressed in μmol TE/L beer (Trolox equivalents, Table 2). Hence, on the one hand, quantitatively dominating constituents in beer and on the other hand structures with an exceptional antioxidant activity in the *in vitro* assays occur with high activity values. In particular, the hordatines (**34**–**36**) as well as the investigated flavan-3-ols (**20**–**23**) showed the highest *in vitro* antioxidant activity as purified compounds. With 13 in the ORAC, 4.7 in the HPS, and 12 μmol TE/μmol in the LA-assay, for **22**, a similar or even higher activity was measured than for **36** with 17.5, 4.1, and 6.1 μmol TE/μmol (ORAC-, HPS- and LA-assay). However, **32** and **33** showed comparably low antioxidant activity, though they exhibited the highest content among the investigated compounds. Consequently, **32** and **33** had the highest activity values in the ORAC assay of 330 and 293 μmol TE/L beer, respectively. With values of 31.1 and 44.3 μmol TE/L beer in the HPS assay, however, the impact was comparable with **22** (33.4 μmol TE/L beer), **23** (45.7 μmol TE/L beer), **8** (37.9 μmol TE/L beer), and **18** (32.1 μmol TE/L beer). In the LA assay, **32** and **33** did not even indicate an activity above 0.01 μmol TE/μmol, leading to the highest activity values for **22** with 84.8, **23** with 79.0, and **18** with 58.2 μmol TE/L beer. Considering all three *in vitro* assays, among all investigated constituents, a group of 10 key antioxidants might be deduced with activity values above 40 in the ORAC, and 8 μmol TE/L beer in the HPS assay, comprising at the same time representatives of the different compound classes. In addition to the amino acids **32** and **33**, the flavan-3-ols **20**, **22**, and **23**, the phenols **6** and **8**, as well as hordatines **34** and **35** and phenylglucoside **18** were found to be key antioxidants in beer. In the order of their activity values, they are followed by homologues **36** and **21**, while further single compounds do not exceed activity values of 20 in the ORAC, 5.5 in the HPS, and 6 μmol TE/L beer in the LA assay.

In further comprehensive studies or routine analysis, these compounds can be used to monitor the antioxidant content of beer. They also cover well the different ingredients and brewing steps, with **6**, **32**, and **33** being derived from brewing malt, as well as **18**, **34**, and **35**, which are partially released from precursors during fermentation along with the appearance in malt, whereas **8** is exclusively generated during fermentation.^15^**20**, **22**, and **23** originate from both barley and hops.^15^ However, antioxidants exclusively derived from hops had comparably low activity values, which is mainly due to their low concentration in beer. Examples are flavonolglucosides **24**–**28**, not exceeding an average concentration 0.65 μmol/L beer, and activity values below 3.5 in the ORAC, 1.5 in the HPS, and 1.5 μmol TE/L beer in the LA-assay. The Maillard compounds **37**–**38** also play only a minor role, mainly due to their low *in vitro* antioxidant activity, although the two analyzed compounds cannot cover the effect of the huge variety of Maillard reaction products, which might still be important due to additive effects.

### Model Experiments for Disclosure of Oxidation Markers in Beer

**Figure 6:**
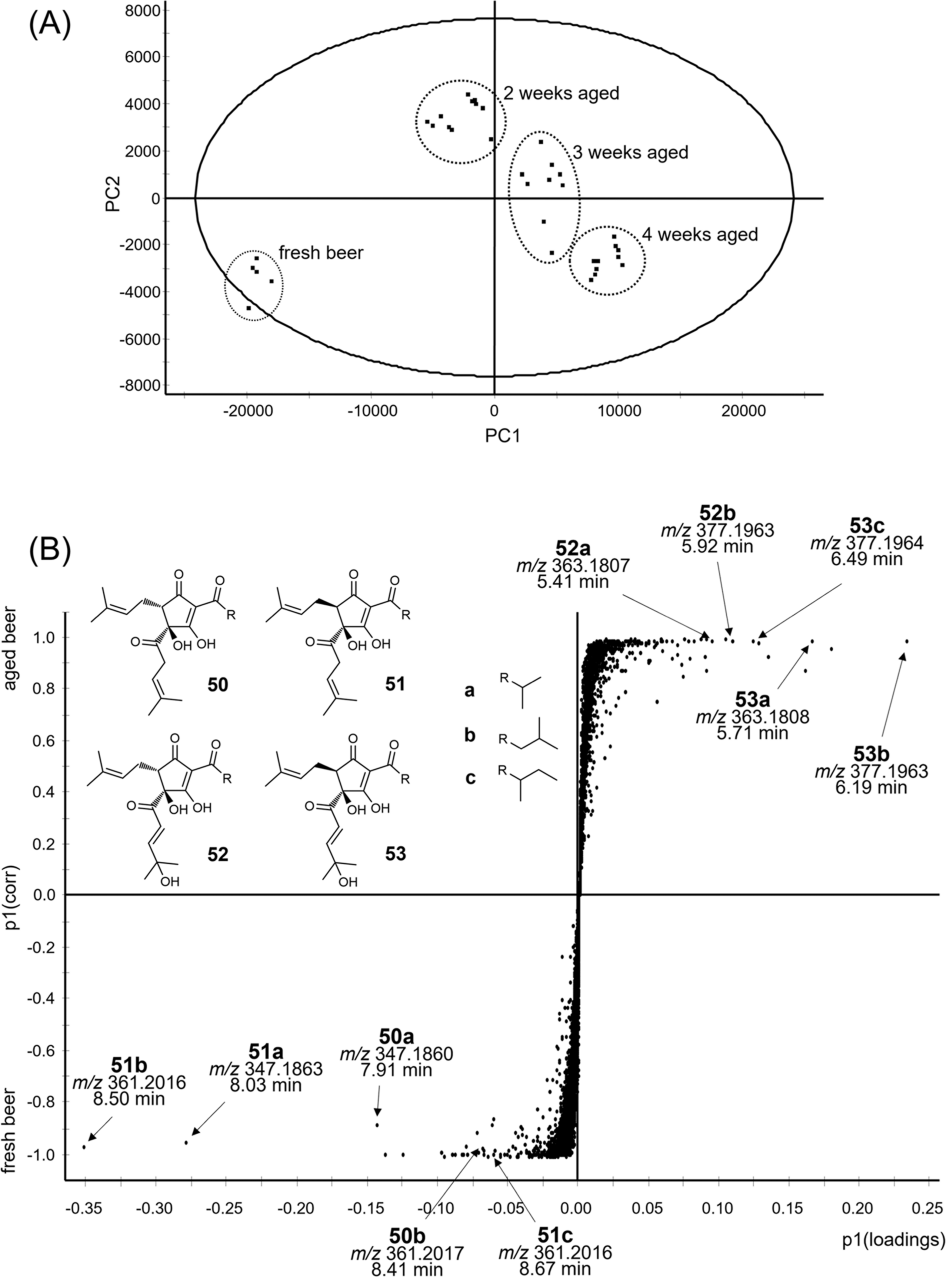
(A) Score plot after PCA of beer samples stored under oxygen atmosphere and investigated by UPLC-TOF-MS, plotting the first two principal components. (B) S-plot calculated after OPLS-DA using the UPLC-TOF-MS data, with the *p*1-value of the loadings on the *x*-axis against the correlation between peak area and classification (fresh beer as −1 and beer aged for 4 weeks as +1) on the *y*-axis, revealing the marker compounds *trans*-isohumulones (**50a**–**c**), *cis-*isohumulones (**51a**–**c**), hydroxyl-*trans-*alloisohumulones (**52a**–**c**), and hydroxyl-*cis-*alloisohumulones (**53a**–**c**).

After their impact was estimated, judged by *in vitro* assays, the effect of antioxidants in pilsner beer as a natural system was also examined. Therefore, it was necessary to find appropriate marker compounds to be able to evaluate the progress of oxidative aging. To reach this aim, beer was stored in two independent batches under an oxygen atmosphere at room temperature for up to 4 weeks, to force oxygen-mediated reactions in this model, and investigated in comparison to a fresh beer using UPLC-TOF-MS. The scores plot after principal component analysis (PCA) revealed a low variation between the two different batches and five technical replicates, confirming good data quality through low analytical variation (Figure 6A). Comparing samples with a different storage time (2, 3, and 4 weeks of storage), however, pointed out significant differences between stored and fresh samples. After classification using OPLS-DA, the results were visualized in an *S*-plot to depict the molecular basis of the changes in composition (Figure 6B).

Thereby, three exceptional markers were observed for fresh beer (p1corr close to −1), with pseudo-molecular ions [M – H]^−^ measured at *m*/*z* 347.1860 [C_20_H_28_O_5_–H]^−^, *m*/*z* 347.1863 [C_20_H_28_O_5_–H]^−^, and *m*/*z* 361.2016 [C_21_H_30_O_5_–H]^−^, indicating isohumulones (**50a**–**c**, **51a**–**c**) with a mass shift of 14 Da being typical for *co-* and *n-*congeners. Isolating references from a commercial iso-α-acid extract and comparing the spectrometric data, co-chromatography confirmed the proposals and led to *trans*-isocohumulone (**50a**), *cis*-isocohumulone (**51a**), and *cis*-isohumulone (**51b**). Additionally, further iso-α-acid congeners were assigned as markers with a lower significance level. Indicators for aged beer (p1corr close to +1, 4 weeks of storage time) revealed pseudo-molecular ions [M – H]^−^ at *m*/*z* 363.1808 [C_20_H_28_O_6_–H]^−^ and particularly *m*/*z* 377.1963 [C_21_H_30_O_6_–H]^−^ with a shift of 16 Da compared to **50a**–**c** and **51a**–**c**, caused by an additional oxygen atom. Proposing hydroxyl-alloisohumulones (**52a**–**c**, **53a**–**c**) as described,^10^ they were generated by oxidizing isohumulones (**50a**–**c**, **51a**–**c**), followed by isolation by means of preparative HPLC, and hydroxyl-*cis*-alloisocohumulone (**53a**) and hydroxyl-*cis*-alloisohumulone (**53b**) were confirmed by co-chromatography. Further congeners were assigned again as markers with a lower significance level, leading to **52a**–**c** and **53a**–**c** as suitable indicators for the oxygen-dependent degradation of **50a**–**c** and **51a**–**c**, being also made plausible by the literature describing the autoxidation mechanism *via* hydroperoxy-alloisohumulones.^10^

### Spiking and Storage Trials with Antioxidants in Beer

After discovering analytical marker compounds for oxidative beer aging, these compounds were further analyzed in comprehensive storage trials after partially increasing the natural content of antioxidants in beer. For those quantitative studies, an optimized chromatographic HPLC-MS/MS method using the ECHO technique was applied^29,35^ (Figure S1). In the storage trials, the natural amounts of antioxidants were doubled, based on the quantitative data of the utilized pilsner-type beer. Thereby, in one series of batches, the concentration of each structural group of antioxidants was increased as another series of batches was spiked continuously with the antioxidants that held the highest activity values (Table 3). The aging conditions were kept equal for all batches, whereby the antioxidants were dissolved in aliquots of degassed beer and overlaid with argon as an inert gas to emulate the natural conditions of a CO_2_-saturated headspace before storing at 40 °C in the dark without stirring.

**Table 3:** Natural and Additionally Spiked Concentration of Antioxidants in the Given Storage Batches

no.^a^	natural conc. [μmol/L]	spiked conc. [μmol/L]	batch
**1**	2.08	2.25	7, F
**2**	4.47	4.48	7, F
**3**	0.33	0.28	7, F
**4**	3.29	3.31	7, F
**5**	0.26	2.32	7, F
**6**	8.87	8.97	6–7, F
**7**	1.15	0.99	7, F
**8**	59.7	59.9	6–7, F
**9**	0.91	0.91	7, F
**10**	3.84	3.75	7, F
**11**	0.12	1.15	7, F
**12**	0.37	0.37	7, F
**13**	1.63	1.59	7, F
**14**	0.89	0.82	7, F
**15**	1.11	1.01	7, F
**16**	1.80	1.83	7, F
**17**	1.39	1.38	7, E
**18**	25.4	25.5	5–7, E
**19a**	3.01	3.07	7, F
**19c**	0.31		
**19b**	0.11		
**20**	6.37	6.23	4–7, D
**21**	2.37	2.39	4–7, D
**22**	5.54	5.51	4–7, D
**23**	6.21	6.12	4–7, D
**24**	0.31	0.30	7, F
**25**	0.23	0.24	7, F
**26**	0.01		
**27**	0.02		
**28**	0.01		
**29**	5.33	5.40	7, F
**30**	0.27	0.27	7, F
**31**	1.18	1.16	7, F
**32**	130	130	1–7, A
**33**	270	269	2–7, B
**34**	9.86	9.77	3–7, C
**35**	10.7	10.7	3–7, C
**36**	1.86	1.83	7, C
**37**	0.43	4.48	7, G
**38**	1.25	1.23	7, G
**39**	>0.02		
**40**	1.00	0.98	7, E
**41**	2.29	2.19	7, E
**42**	0.26	0.26	7, C
**43**	0.37	0.37	7, C
**44**	0.03	0.03	7, C
**50a**	13.2	13.2	H
**51a**	31.8	31.8	H

a Chemical structures given in Figure 1.

**Figure 7:**
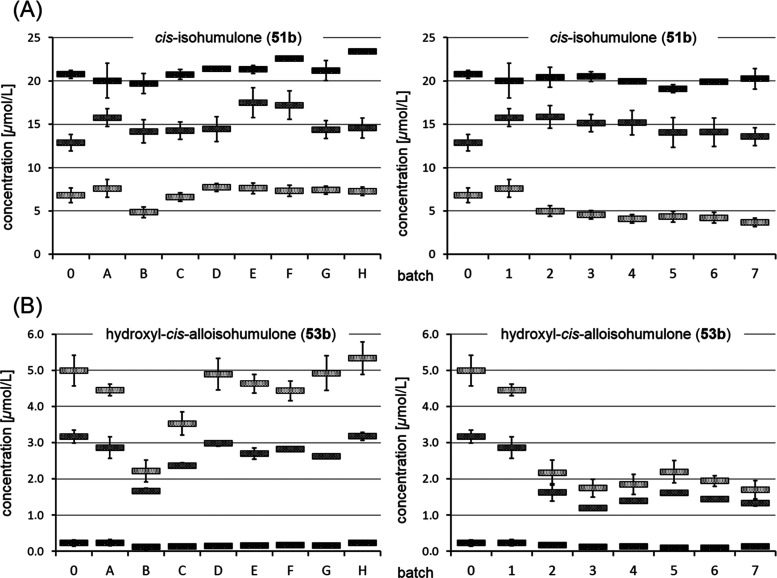
Concentrations of **51b** (A) and **53b** (B) in beer samples spiked with antioxidants (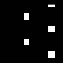) and then stored at 40 °C for 7 days (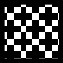) and 21 days (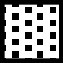). Numbering and constituents of batches are given in Table 3.

Exemplarily for **50a**–**c** to **51a**–**c** and **52a**–**c** to **53a**–**c**, each quantitatively dominating *cis-n-*congener (**51b**, **53b**) was investigated by HPLC-MS/MS (Figure 7). The concentration of **51b** behaved similarly in all batches, with about 20 μmol/L in the fresh samples decreasing steadily throughout 3 weeks of storage to 5 μmol/L (Figure 7A). After a storage time of just 1 week, slight differences suggested an aging slow down through the addition of antioxidants, as the lowest content of the freshness marker was measured for the unspiked sample with 12.9 μmol/L. However, the levels equalized after 3 weeks of storage, which might indicate that the degradation reached an end point that cannot be modulated by antioxidants. Apart from that, further non-oxygen-mediated reactions that are not impacted by antioxidants have to be taken into consideration, such as published pH-driven cycling reactions of the *trans*-congeners.^9^

In contrast, observing the evolution of the amounts of **53b** highlighted the importance of antioxidants (Figure 7B). In the unspiked sample, the concentration rose to 5.0 μmol/L within 3 weeks of storage, which was not affected by an additional amount of **50a** and **51a** in batch H, investigating the effect of a higher total iso-α-acid content. In the same way, the Maillard compounds **37**–**38** in batch G had no inhibiting effect, as already expected on the basis of their activity values, though they cannot be used to judge the impact of all Maillard reaction products in beer, including melanoidins. Surprisingly, the flavan-3-ols **20**–**23** in batch D also did not show an inhibiting effect, and the numerous phenolics in batch F indicated just a slight effect on the aging reaction, as 4.4 μmol/L were recorded in comparison to 5.0 μmol/L in the unspiked sample. The compounds of batch E with 4.6 μmol/L and batch A, containing **32**, with 4.5 μmol/L also just led to a slowdown of about 10%, giving a hint on an activity in beer. However, **33** (batch B) and **34**–**36** (batch C) revealed the strongest and highly significant effect on the inhibition of **53b**, with a rate of 56, and 29%, respectively. Equally, the subsequent addition of antioxidants in batches 1–7 led to a steady decrease in the yield of the degradation product, especially after the addition of **33**–**35** in batches 3–7, confirming their central role in these studies. Thereby, it appears that a maximum possible inhibition was already reached, as an enrichment with further antioxidants had no significant effect, although prooxidative effects have not been observed either. Summarizing, doubling the natural amounts of all investigated antioxidants led to an inhibition of 67%, as a value of 1.7 μmol/L **53b** was measured as compared to 5.0 μmol/L in the unspiked sample. In summary, these results demonstrate a decelerating effect of antioxidants on oxygen-dependent aging reactions occurring during the storage of beer and accordingly suggest a positive effect on the flavor stability of beer, even at naturally relevant levels.

## Supporting Information Available

Mass transitions and optimized ionization parameters of the utilized quantitation techniques and detailed quantitative data of the investigated beer samples (PDF)
