# Supplementary material for: Quantitation of Key Antioxidants and Their Contribution to the Oxidative Stability of Beer
Source: J Agric Food Chem. 2024 Jul 16;72(29):16423–37. doi: 10.1021/acs.jafc.4c01000 (PMC11273605; doi:10.1021/acs.jafc.4c01000)
Supplement: Supplementary file 1 — jf4c01000_si_001.pdf [file jf4c01000_si_001.pdf]

Supporting Information

Quantitation of Key-Antioxidants and Their Contribution to the Oxidative Stability of Beer

Stefan Spreng[1], Corinna Dawid[1,2], Andreas Dunkel[3], and Thomas Hofmann[1,2*]

[1]Chair of Food Chemistry and Molecular and Sensory Science, Technical University of Munich, Lise-Meitner-Str. 34, D-85354 Freising, Germany, [2]Bavarian Center for Biomolecular Mass Spectrometry, Gregor-Mendel-Straße 4, D-85354 Freising, Germany, and [3]Leibniz-Institute for Food Systems Biology at the Technical University of Munich, Lise-Meitner Str. 34, D-85354 Freising, Germany.

---

[*] **To whom correspondence should be addressed**

PHONE +49-8161/71-2902

FAX +49-8161/71-2949

E-MAIL thomas.hofmann@tum.de

Figure S1. HPLC-MS/MS analysis of a beer sample showing the mass transition traces of the ageing indicators 50−53. Signal intensity of each mass transition is normalized and numbering of compounds refers to chemical structures given in Figure 6, as ECHO standards are marked with an "S".

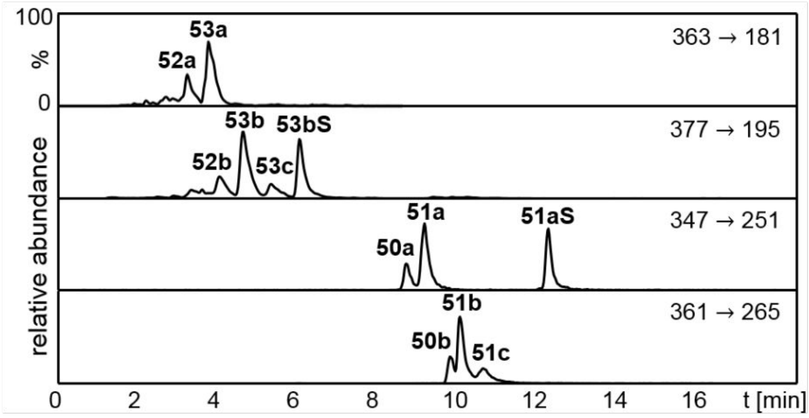

Table S1. Overview of the Investigated Commercial Beer Samples.

No.	Name	ABV [a]	OE [b]	Group	Fermentation
I	Bitburger Pils	4.8 %	11.3	pilsner beer	bottom-fermented
II	Jever Pils	4.9 %	11.3	pilsner beer	bottom-fermented
III	Beck's Pils	4.9 %	11.2	pilsner beer	bottom-fermented
IV	Pilsener Urquell	4.4 %	11.8	pilsner beer	bottom-fermented
V	Paulaner Münchner Hell	4.9 %	11.5	pale lager	bottom-fermented
VI	König Ludwig Hell	5.1 %	11.4	pale lager	bottom-fermented
VII	Augustiner Lagerbier Hell	5.2 %	11.5	pale lager	bottom-fermented
VIII	Löwenbräu Hell	5.2 %	11.8	pale lager	bottom-fermented
IX	Herrnbräu Hell	4.8 %	11.6	pale lager	bottom-fermented
X	König Ludwig Dunkel	5.1 %	12.0	dark lager	bottom-fermented
XI	Köstritzer Schwarzbier	4.8 %	11.4	dark lager	bottom-fermented
XII	Paulaner Weißbier Hell	5.5 %	12.5	pale wheat beer	top-fermented
XIII	Weihenstephaner Weißbier Hell	5.4 %	12.7	pale wheat beer	top-fermented
XIV	Erdinger Weißbier	5.3 %	12.6	pale wheat beer	top-fermented
XV	Erdinger Urweisse	4.9 %	11.9	pale wheat beer	top-fermented
XVI	Paulaner Weißbier Dunkel	5.3 %	12.4	dark wheat beer	top-fermented
XVII	Weihenstephaner Weißbier Dunkel	5.3 %	12.7	dark wheat beer	top-fermented

XVIII	Paulaner Salvator Starkbier	7.9 %	18.3	special-type beer (strong beer)	bottom-fermented
XIX	Weltenburger Barock Dunkel	4.7 %	12.5	special-type beer (Munich dark)	bottom-fermented
XX	Guiness Extra Stout	4.1 %	9.8	special-type beer (stout)	top-fermented
XXI	Fuller's London Pride Pale Ale	4.7 %	N/A	pale ale	top-fermented
XXII	Beck's Pale Ale	6.3 %	15.0	pale ale	top-fermented
XXIII	Camba German India Pale Ale	6.5 %	15.5	pale ale (India pale ale)	top-fermented

[a] alcohol content by volume (ABV) given in % as specified by the manufacturer; [b] original extract (OE) of the wort given in °P as specified by the manufacturer.

Table S2. Monitored Mass Transitions and Optimized MS/MS Parameters of the Quantitative Analysis of Antioxidants in ESI$^-$ Mode.

No. [a]	Q1	→	Q3	qn/ql [b]	DP [c]	EP [d]	CE [e]	CXP [f]
**8**	137.0	→	106.0	qn	−65	−10	−22	−7
**8**	137.0	→	118.8	ql	−65	−10	−22	−21
**1**	136.9	→	92.8	qn	−50	−10	−22	−7
**1**	136.9	→	64.9	ql	−50	−10	−42	−9
**4, 47**	163.0	→	119.0	qn	−55	−10	−20	−1
**4, 47**	163.0	→	92.8	ql	−55	−10	−44	−5
**2**	166.9	→	108.0	qn	−55	−10	−26	−5
**2**	166.9	→	123.1	ql	−55	−10	−18	−1
**5**	179.0	→	135.0	qn	−50	−10	−22	−9
**5**	179.0	→	107.0	ql	−50	−10	−32	−7
**37**	180.0	→	93.9	qn	−45	−10	−24	−5
**37**	180.0	→	84.9	ql	−45	−10	−14	−7
**9**	181.0	→	162.7	qn	−60	−10	−18	−11
**9**	181.0	→	134.9	ql	−60	−10	−24	−7
**6**	193.0	→	133.8	qn	−55	−10	−22	−7
**6**	193.0	→	177.9	ql	−55	−10	−18	−9
**3**	197.0	→	181.9	qn	−60	−10	−18	−11
**3**	197.0	→	122.9	ql	−60	−10	−34	−1
**38**	210.0	→	123.9	qn	−60	−10	−22	−1
**38**	210.0	→	93.8	ql	−60	−10	−32	−7
**46**	211.0	→	136.8	qn	−55	−10	−32	−7
**46**	211.0	→	166.8	ql	−55	−10	−18	−11
**7**	223.0	→	163.9	qn	−60	−10	−20	−9
**7**	223.0	→	120.9	ql	−60	−10	−40	−5
**49**	269.1	→	117.1	qn	−85	−10	−48	−1
**49**	269.1	→	150.9	ql	−85	−10	−34	−7
**17**	271.0	→	160.7	qn	−85	−10	−12	−33
**17**	271.0	→	107.6	ql	−85	−10	−32	−15
**45**	285.1	→	160.7	qn	−80	−10	−12	−27
**45**	285.1	→	107.9	ql	−80	−10	−66	−25

**20, 21**	289.0	→	245.1	qn	−75	−10	−22	−7
**20, 21**	289.0	→	108.8	ql	−80	−10	−40	−7
**18**	301.1	→	160.7	qn	−55	−10	−12	−9
**18**	301.1	→	122.7	ql	−55	−10	−44	−7
**48**	303.0	→	284.8	qn	−60	−10	−14	−17
**48**	303.0	→	174.6	ql	−60	−10	−24	−11
**11**	312.1	→	147.8	qn	−95	−10	−36	−7
**11**	312.1	→	178.0	ql	−95	−10	−30	−11
**29, 30**	353.1	→	118.8	qn	−110	−10	−42	−9
**29, 30**	353.1	→	232.9	ql	−110	−10	−42	−9
**12**	357.0	→	150.7	qn	−100	−10	−26	−9
**12**	357.0	→	135.7	ql	−100	−10	−48	−7
**19a**	357.1	→	194.8	qn	−100	−10	−24	−11
**19a**	357.1	→	150.9	ql	−100	−10	−48	−9
**16**	367.1	→	192.9	qn	−65	−10	−24	−3
**14**	367.1	→	190.8	qn	−65	−10	−24	−3
**15**	367.1	→	172.8	qn	−65	−10	−22	−3
**19b, 19c**	371.1	→	208.8	qn	−100	−10	−24	−11
**19b, 19c**	371.1	→	164.9	ql	−100	−10	−48	−9
**13**	417.1	→	180.7	qn	−85	−10	−28	−11
**13**	417.1	→	165.7	ql	−85	−10	−46	−9
**24**	447.1	→	283.8	qn	−105	−10	−38	−17
**24**	447.1	→	254.8	ql	−105	−10	−54	−15
**25**	463.1	→	300.0	qn	−100	−10	−36	−19
**25**	463.1	→	271.0	ql	−100	−10	−62	−17
**41**	463.2	→	323.1	qn	−160	−10	−20	−11
**41**	463.2	→	178.9	ql	−160	−10	−24	−11
**26**	477.1	→	314.1	qn	−105	−10	−38	−9
**26**	477.1	→	243.1	ql	−105	−10	−56	−15
**27**	533.2	→	489.2	qn	−50	−10	−18	−23
**27**	533.2	→	285.1	ql	−50	−10	−36	−19
**28**	549.2	→	505.2	qn	−55	−10	−18	−23
**28**	549.2	→	299.9	ql	−55	−10	−46	−7
**22**	577.3	→	406.9	qn	−80	−10	−26	−19

**22**	577.3	→	288.9	ql	−80	−10	−26	−9
**23**	593.2	→	289.1	qn	−95	−10	−36	−11
**23**	593.2	→	407.2	ql	−95	−10	−38	−7
**31**	593.2	→	311.0	qn	−180	−10	−50	−15
**31**	593.2	→	296.9	ql	−180	−10	−62	−13
**39**	595.2	→	178.9	qn	−150	−10	−36	−11
**39**	595.2	→	220.9	ql	−150	−10	−42	−1
**40**	625.3	→	179.0	qn	−155	−10	−36	−11
**40**	625.3	→	221.1	ql	−155	−10	−42	−1

[a] chemical structures given in Figure 1 and 2; [b] qn: quantifier mass transition, ql: qualifier mass transition; [c] Declustering Potential [V]; [d] Entrance Potential [V]; [e] Collision Energy [V]; [f] Collision Cell Exit Potential [V].

Table S3. Monitored Mass Transitions and Optimized MS/MS Parameters of the Quantitative Analysis of Antioxidants in ESI$^+$ Mode.

No. [a]	Q1	→	Q3	qn/ql [b]	DP [c]	EP [d]	CEP [e]	CE [f]	CXP [g]
**33**	182.2	→	136.0	qn	21	11.5	17	21	4
**33**	182.2	→	91.1	ql	21	11.5	17	39	4
**33a**	186.0	→	127.0	qn	38	10	17	25	10
**33a**	186.0	→	140.0	ql	38	10	17	19	10
**32**	205.2	→	146.0	qn	31	8.5	16	15	4
**32**	205.2	→	114.9	ql	31	5.5	16	25	4
**32a**	210.0	→	150.0	qn	40	10	18	26	10
**32a**	210.0	→	122.0	ql	40	10	18	37	10
**10**	261.0	→	136.0	qn	41	4	20	28	3
**10**	261.0	→	120.0	ql	37	9	20	29	3
**34**	276.3	→	72.2	qn	31	7.5	32	41	4
**34**	276.3	→	98.0	ql	31	7.5	32	25	4
**35**	291.2	→	131.2	qn	31	7.5	24	27	4
**35**	291.2	→	72.1	ql	31	7.5	24	47	4
**36**	306.3	→	131.0	qn	31	7.5	22	27	4
**36**	306.3	→	71.8	ql	31	7.5	22	57	4
**42**	357.4	→	276.2	qn	6	11.5	22	25	2
**42**	357.4	→	72.0	ql	6	11.5	22	59	4
**43**	372.4	→	291.2	qn	11	7	24	25	4
**43**	372.4	→	72.2	ql	11	7	24	57	4
**44**	387.4	→	306.2	qn	6	10.5	22	25	6
**44**	387.4	→	72.1	ql	6	10.5	22	59	4

[a] chemical structures given in Figure 1 and 2; [b] qn: quantifier mass transition, ql: qualifier mass transition; [c] Declustering Potential [V]; [d] Entrance Potential [V]; [e] Collision Cell Entrance Potential [V]; [f] Collision Energy [V]; [g] Collision Cell Exit Potential [V].

Table S4. Monitored Mass Transitions and Optimized MS/MS Parameters of the Quantitative Analysis of Ageing Markers.

No. [a]	Q1		Q3	qn/ql [b]	DP [c]	EP [d]	CE [e]	CXP [f]
**50a, 51a**	347.1	→	251.1	qn	−65	−10	−22	−15
**50a, 51a**	347.1	→	110.9	ql	−65	−10	−64	−5
**50b, 50c, 51b, 51c**	361.2	→	265.1	qn	−95	−10	−24	−7
**50b, 50c, 51b, 51c**	361.2	→	195.8	ql	−105	−10	−32	−13
**52a, 53a**	363.3	→	180.9	qn	−110	−10	−44	−13
**52a, 53a**	363.3	→	110.6	ql	−110	−10	−60	−7
**52b, 52c, 53b, 53c**	377.3	→	194.9	qn	−110	−10	−44	−13
**52b, 52c, 53b, 53c**	377.3	→	124.6	ql	−110	−10	−60	−7

[a] chemical structures given in Figure 1 and 2; [b] qn: quantifier mass transition, ql: qualifier mass transition; [c] Declustering Potential [V]; [d] Entrance Potential [V]; [e] Collision Energy [V]; [f] Collision Cell Exit Potential [V].

Table S5. Concentration of Beer Antioxidants in Commercial Beer Samples in μmol/L.

No.[a]	pilsner				pale lager					dark lager	
	I	II	III	IV	V	VI	VII	VIII	IX	X	XI
**1**	2.08	2.12	2.25	1.84	2.18	1.35	1.27	2.43	1.99	1.31	1.88
**2**	4.47	3.13	2.30	4.58	3.63	3.89	3.38	2.20	3.78	5.48	4.13
**3**	0.33	0.34	0.32	0.42	0.36	0.31	0.40	0.38	0.34	0.53	0.44
**4**	3.29	6.13	2.32	6.18	4.40	3.03	1.97	2.59	2.56	5.25	5.19
**5**	0.26	0.28	0.21	0.42	0.25	0.18	0.18	0.21	0.20	0.22	0.30
**6**	8.87	11.9	7.05	18.3	10.5	7.96	6.83	8.27	7.35	8.77	10.3
**7**	1.15	1.26	1.19	1.94	1.20	1.06	1.32	1.50	1.17	1.07	1.38
**8**	59.7	40.2	51.6	45.2	39.4	43.7	78.8	46.2	55.5	56.1	43.9
**9**	0.91	1.00	0.87	0.83	1.06	0.98	0.76	1.09	1.00	1.31	1.06
**10**	3.84	3.78	3.96	3.32	3.45	2.95	2.80	4.04	1.98	4.71	5.91
**11**	0.12	0.06	0.13	0.35	0.09	0.13	0.14	0.08	0.13	0.08	0.11
**12**	0.37	0.40	0.50	0.52	0.39	0.40	0.34	0.38	0.35	0.43	0.35
**13**	1.63	1.47	1.38	1.71	1.71	1.40	1.47	2.05	1.33	1.46	1.52
**14**	0.89	0.46	0.61	0.66	0.68	0.45	0.43	0.53	0.57	0.33	0.48
**15**	1.11	0.95	1.34	0.95	0.90	0.76	1.02	1.04	1.08	0.77	0.69
**16**	1.80	1.41	2.40	1.51	1.44	1.29	1.58	1.84	1.63	1.38	1.13
**17**	1.39	1.52	1.54	1.47	1.95	1.94	1.56	1.87	1.55	1.66	1.73
**18**	25.4	34.7	33.2	28.5	36.7	32.2	31.8	37.5	34.0	33.1	29.2
**19a**	3.01	0.33	6.59	7.52	7.00	1.31	2.34	1.98	3.52	1.65	1.45
**19c**	0.11	< 0.02	0.26	0.34	0.26	0.06	0.09	0.06	0.09	0.08	0.03
**19b**	0.31	0.03	0.58	0.89	0.72	0.14	0.21	0.18	0.26	0.16	0.12
**20**	6.37	3.84	2.54	5.15	3.78	10.1	12.1	2.33	6.16	7.67	6.15
**21**	2.37	1.79	1.25	2.35	2.10	2.72	2.91	1.01	2.55	1.36	1.92
**22**	5.54	3.89	4.02	4.95	6.15	15.6	18.9	5.09	12.3	7.44	6.83
**23**	6.21	4.33	4.30	3.06	7.47	21.8	26.2	7.70	13.7	11.2	6.60
**24**	0.31	0.03	0.74	1.24	0.43	0.17	0.70	0.29	0.40	0.15	0.20
**25**	0.23	0.03	0.64	1.28	0.45	0.19	0.79	0.28	0.39	0.19	0.23

**26**	0.01	0.004	0.02	0.03	0.02	0.01	0.02	0.01	0.01	0.004	0.01
**27**	0.02	0.03	0.23	0.94	0.18	0.04	0.21	0.12	0.19	0.10	< 0.02
**28**	< 0.02	< 0.02	0.31	1.47	0.21	0.05	0.34	0.14	0.32	0.13	0.02
**29**	5.33	0.31	4.51	6.76	3.59	4.65	3.53	1.52	3.29	1.68	7.53
**30**	0.27	0.04	0.15	0.76	0.33	0.83	0.42	0.17	0.28	3.20	5.41
**31**	1.18	1.08	1.23	1.14	1.64	1.35	1.88	1.43	1.74	1.27	1.34
**32**	130	188	124	184	153	176	154	179	146	135	150
**33**	270	338	225	393	273	313	254	388	188	291	287
**34**	9.86	11.2	10.5	5.16	8.82	10.9	12.6	14.3	11.8	13.0	9.87
**35**	10.7	8.56	6.79	4.78	6.39	7.63	12.8	9.66	9.82	8.06	7.74
**36**	1.86	1.57	1.55	1.25	1.08	1.20	2.12	1.91	1.71	1.60	1.66
**37**	0.43	0.62	0.37	0.47	0.41	0.38	0.32	1.01	0.64	2.04	0.71
**38**	1.25	1.94	1.17	3.76	1.01	1.33	0.93	3.34	1.02	11.5	4.87
**39**	< 0.07	< 0.07	< 0.07	< 0.07	< 0.07	< 0.07	< 0.07	< 0.07	< 0.07	< 0.07	< 0.02
**40**	1.00	1.44	0.91	1.68	1.14	0.94	0.86	1.34	0.60	1.31	0.77
**41**	2.29	3.14	1.85	3.06	3.16	2.35	1.53	1.72	1.34	2.71	2.39
**42**	0.26	1.73	2.25	2.52	1.10	0.84	0.54	4.82	0.65	3.74	1.36
**43**	0.37	5.12	6.48	8.86	3.69	2.96	3.38	11.3	3.01	8.52	4.74
**44**	0.03	0.02	0.02	0.03	0.02	0.01	0.02	0.05	0.02	0.01	0.03

[a] Chemical structures are given in Figure 1.

Table S6. Concentration of Beer Antioxidants in Commercial Beer Samples in μmol/L.

No.[a]	pale wheat beer				dark wheat beer		special-type beer			pale ale		
	XII	XIII	XIV	XV	XVI	XVII	XVIII	XIX	XX	XXI	XXII	XXIII
**1**	1.47	1.18	1.23	1.28	1.82	1.74	3.15	3.03	3.25	2.07	1.80	5.73
**2**	2.64	3.02	2.60	3.07	2.68	1.83	7.69	3.46	1.81	2.51	1.41	4.28
**3**	0.81	0.73	0.68	0.67	0.76	0.83	0.63	0.51	0.29	0.36	0.40	0.61
**4**	< 0.09	< 0.09	6.47	< 0.09	< 0.09	< 0.09	8.55	4.90	1.87	1.98	3.48	5.30
**5**	0.26	0.19	0.64	0.25	0.31	0.50	0.44	0.39	0.20	0.41	0.29	0.92
**6**	0.36	0.31	14.3	0.31	0.37	0.41	17.2	11.5	4.78	6.97	11.5	13.5
**7**	1.55	1.45	2.00	1.49	1.40	1.73	1.55	0.93	0.46	0.91	1.74	2.11
**8**	54.5	29.8	48.5	33.5	49.9	39.6	64.9	54.9	17.1	15.1	126	20.4
**9**	1.83	0.89	1.88	1.31	1.05	1.14	1.83	1.27	1.27	1.51	1.76	0.91
**10**	2.22	2.28	2.81	2.44	3.22	3.31	5.37	3.81	8.68	5.23	4.86	4.05
**11**	0.02	0.04	0.08	0.06	0.05	0.06	0.17	0.12	0.05	0.18	0.13	0.58
**12**	0.22	0.22	0.28	0.24	0.21	0.25	0.51	0.42	0.23	0.54	0.58	1.06
**13**	2.51	2.18	2.75	2.44	2.38	2.81	2.60	1.49	0.98	1.38	1.69	2.03
**14**	0.57	0.69	0.71	0.36	0.67	0.56	1.07	0.48	0.36	0.63	0.56	1.18
**15**	0.88	0.80	0.93	0.60	0.74	0.68	1.40	0.95	0.68	1.34	1.45	2.97
**16**	1.12	1.13	1.44	0.86	1.18	1.11	2.14	1.73	0.98	2.59	2.82	8.78
**17**	1.50	1.57	1.40	1.47	1.43	1.44	2.89	1.98	0.90	1.38	2.05	2.44
**18**	29.5	25.5	36.2	29.4	30.3	30.2	55.2	42.4	22.3	23.2	40.5	35.0
**19a**	0.03	1.88	3.10	2.23	0.14	1.36	5.36	7.65	< 0.005	7.92	5.80	32.5
**19c**	< 0.005	0.04	0.09	0.06	< 0.02	0.05	0.17	0.30	< 0.005	0.29	0.16	1.58
**19b**	< 0.005	0.14	0.27	0.19	0.02	0.11	0.51	0.78	< 0.005	0.72	0.41	3.79
**20**	3.19	3.51	4.52	5.32	2.41	3.89	5.89	4.46	5.63	6.12	2.62	30.9
**21**	1.03	1.19	1.44	1.66	0.94	1.03	1.90	1.15	1.07	2.10	1.39	8.63
**22**	1.95	3.00	3.98	6.91	1.15	2.07	2.00	4.35	5.94	2.34	3.59	35.7
**23**	2.96	2.96	4.04	9.09	1.44	2.35	1.84	4.82	8.01	2.19	2.75	30.3
**24**	0.01	0.12	0.32	0.19	0.01	0.12	0.52	0.62	0.04	0.74	0.76	5.40
**25**	< 0.02	0.17	0.34	0.16	0.02	0.16	0.57	0.75	0.03	0.87	0.65	6.04

**26**	< 0.002	0.004	0.01	0.01	< 0.002	0.01	0.02	0.02	0.004	0.03	0.02	0.15
**27**	< 0.01	0.04	0.03	0.09	< 0.01	< 0.02	0.04	0.31	< 0.02	0.25	0.50	3.94
**28**	< 0.01	0.07	0.04	0.09	< 0.01	0.02	0.06	0.56	< 0.01	0.56	0.62	6.18
**29**	0.49	3.44	3.48	2.37	3.17	2.59	8.11	6.78	0.05	3.45	4.74	8.42
**30**	0.10	0.59	0.46	0.66	0.89	0.97	0.65	3.89	0.06	0.51	0.49	5.08
**31**	0.60	0.71	0.71	0.66	0.81	0.64	2.59	1.20	0.64	1.12	1.02	1.98
**32**	159	201	157	209	136	156	175	104	140	168	107	270
**33**	216	271	252	306	181	231	438	233	286	508	128	576
**34**	4.50	5.56	7.65	8.34	4.99	6.93	13.8	11.3	12.3	16.8	9.15	7.00
**35**	3.11	4.25	6.16	6.25	3.14	5.55	9.43	7.30	10.2	14.9	5.28	6.16
**36**	0.62	0.76	1.14	0.99	0.56	0.86	1.70	1.40	2.34	2.61	1.63	0.97
**37**	1.07	0.77	0.96	1.34	1.09	0.97	1.98	2.49	0.42	1.25	0.88	1.62
**38**	5.71	3.00	5.63	14.9	5.33	3.61	11.7	19.7	1.60	7.64	3.58	13.3
**39**	< 0.07	< 0.07	< 0.07	< 0.07	< 0.07	< 0.02	0.11	0.09	0.09	< 0.07	< 0.07	< 0.07
**40**	0.64	0.71	1.06	0.87	0.83	0.75	1.54	1.73	2.99	0.87	2.79	1.47
**41**	1.59	2.28	2.08	2.27	1.59	1.77	3.63	2.88	1.83	1.16	1.57	2.66
**42**	0.92	0.50	1.28	0.88	1.04	0.35	2.06	2.69	4.49	0.55	4.92	0.20
**43**	2.26	1.58	3.71	2.84	2.48	1.13	7.55	7.05	8.21	0.55	9.33	0.22
**44**	0.01	0.01	0.01	0.01	0.01	0.01	0.05	0.02	0.11	0.03	0.06	0.01

[a] Chemical structures are given in Figure 1.
